# The agglomeration and dispersion dichotomy of human settlements on Earth

**DOI:** 10.1038/s41598-021-02743-9

**Published:** 2021-12-02

**Authors:** Emanuele Strano, Filippo Simini, Marco De Nadai, Thomas Esch, Mattia Marconcini

**Affiliations:** 1 MindEarth, 2502 Biel/Bienne, Switzerland; 2 University of Bristol, Bristol, 06010 UK; 3 Fondazione Bruno Kessler (FBK), Trento, Italy; 4 German Aerospace Center (DLR), Wessling, Germany; 5 Argonne Leadership Computing Facility, Argonne National Laboratory, Lemont, IL USA

**Keywords:** Sustainability, Environmental impact, Environmental impact, Statistical physics

## Abstract

Human settlements on Earth are scattered in a multitude of shapes, sizes and spatial arrangements. These patterns are often not random but a result of complex geographical, cultural, economic and historical processes that have profound human and ecological impacts. However, little is known about the global distribution of these patterns and the spatial forces that creates them. This study analyses human settlements from high-resolution satellite imagery and provides a global classification of spatial patterns. We find two emerging classes, namely agglomeration and dispersion. In the former, settlements are fewer than expected based on the predictions of scaling theory, while an unexpectedly high number of settlements characterizes the latter. To explain the observed spatial patterns, we propose a model that combines two agglomeration forces and simulates human settlements’ historical growth. Our results show that our model accurately matches the observed global classification (F1: 0.73), helps to understand and estimate the growth of human settlements and, in turn, the distribution and physical dynamics of all human settlements on Earth, from small villages to cities.

## Introduction

The growth and expansion of cities on Earth influence all global social, economic and environmental systems^1–5^. Abundant evidence indicates that cities have significant impacts on the water and ecological systems, land-use competition, food production, biodiversity, climate change and human health^6–11^, and extensive debates highlight the trade-off between benefits and challenges for global urbanization^12–17^. However, the real extent, distribution and explanation of human settlements (HSs) are not yet fully understood at the global scale, especially regarding the spatial arrangement and type of patterns for settlements of *all sizes*, ranging from vast metropolitan areas to small and scattered rural settlements.

Several factors have hampered a global analysis and description of HSs: on the one hand, quantitative analyses of HSs patterns often rely on traditional spatial metrics used in urban geography^18^, typically extracted from census data^19–22^, and statistical analyses derived from complex systems such as fractals and urban scaling^23^, which are observed only at large spatial scales such as continents and countries. On the other hand, most early studies relied on low- or medium-resolution satellite data that range from 0.5 to 1 km^15,24–27^, which are usually focused on *urban* land cover and thus exclude from the analysis the vast majority of *non-urban* settlements. Although high-resolution global HSs inventories have recently been proposed^28,29^, significant inaccuracies still exist^30^, probably due to the technical challenges of having a uniform and consistently cross-validated global dataset.

Here, we provide an unprecedented global estimation of the geography of HSs by quantitatively analyzing their location, distribution and spatial patterns through the urban scaling based on the Zipf’s law^31–34^. First, we provide a comprehensive global analysis of the location and density of all HSs by exploiting the World Settlement Footprint 2015 (WSF2015)^35^ dataset, an accurate 10 m resolution inventory of human-occupied land. Second, we exploit scaling theory and analyze the deviations from the scale-free distribution of settlement sizes. We discover that in all continents two distinct types of HS patterns emerge: dispersed and agglomerated settlements. These two patterns drive the high heterogeneity of HSs and help understand urbanization in different areas of the world. Finally, we build a minimal spatially explicit model that can reproduce all observed settlement patterns on Earth by inter-playing two agglomeration forces.

## Results

We study HSs on Earth through the WSF2015 dataset^35^, a novel 10m resolution binary mask outlining the human-occupied land in the world. The dataset has been created by jointly exploiting multi-temporal radar (Sentinel-1) and optical (Landsat-8) satellite imagery, and it has been validated extensively^35^ through a collaboration between Google and DLR.

**Figure 1:**
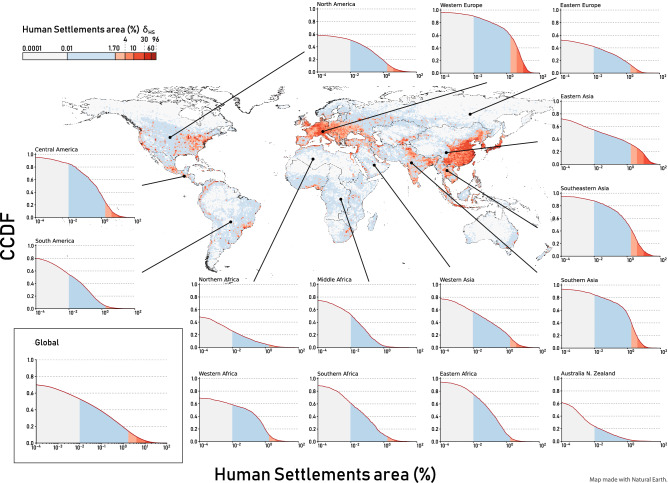
A global overview of HSs density in 2015. For each tile, we compute the percentage of occupied HS area in each tile $\delta _{HS}$ and find that, on average, HSs cover 1.70% of the tile area. Inset: the long-tail distribution of the cumulative frequency of the percentage of occupied HS area within each tile, $\delta _{HS}$. A small number of tiles contains the majority of the settlements. Map: we colour in cyan the tiles with $\delta _{HS}$ less than the global average density ($1.70\%$), while we colour with a red gradient the areas with a density between $1.70\%$ and $100\%$. High-density tiles are not evenly distributed in the world. Small plots: we show how the global long-tail emerge in all the UN-defined macro-areas.

The WSF2015 classifies as human-occupied land a $10 \times 10$ m cell that contains either a building or a building lot, where: (i) a building is any structure having a roof supported by columns or walls and intended for the shelter, housing, or enclosure of any individual, animal, process, equipment, goods, or materials of any kind; and (ii) a building lot is the area contained within an enclosure (e.g., wall, fence, hedge) surrounding a building or a group of buildings. Such an accurate inventory of human presence on Earth allows us to perform an unprecedented analysis of the real magnitude, geography and spatial structure of HSs at the global level.

From the WSF2015, we define an HS as a continuous areas of human-occupied land formed by aggregating neighbouring pixels whenever one touches the other along its edges (see the Methods Section for details). Thus, an HS might be as small as a single building or big as an entire city.

We estimate that the total number of HSs is approximately 32 million and the corresponding area amounts to 1,302,187 km$^2$ (i.e., about $1.04\%$ of the global land surface area estimated in 131,331,424 km$^2$ excluding the Arctic and Antarctic regions). However, not all dry-land surfaces can be settled. Thus, from satellite imagery, we exclude areas with complex topography that are not suitable for hosting HSs (e.g. areas with extremely elevated steepness) and internal freshwater surfaces through a *relief mask* and a *freshwater mask*, respectively (see the Methods and Supporting Information (SI) sections for details). The area of habitable land amounts to 106,445,525 km$^2$; out of this, we estimate that HSs cover $1.22\%$ of the entire world.

However, settlements on Earth are not evenly distributed across regions, and they are very heterogeneous in size and shape. To study such variations, we subdivided the Earth’s surface into 29,181 tiles of $0.5^{\circ } \times 0.5^{\circ }$ (approximately $55 \times 55$ km$^2$ at the equator). We measured the percentage of occupied HS area $\delta _{HS}$, or density, in each tile as the ratio between the tile’s HS area and its total surface area minus the exclusion mask (defined as the combination of the relief and freshwater areas) and find that, on average, HSs occupy 1.70% of the tile’s area. Figure 1 shows the spatial distribution and cumulative frequency of $\delta _{HS}$ at the global scale, and for the 16 macro-areas defined by the United Nations^36^. In the bottom-left inset of Fig. 1 we plotted cumulative frequency at the global scale by fixing on the *x*-axis seven HS percentage thresholds. We find that the density of HSs areas on Earth has a long-tail distribution, which means that a small number of tiles contains the majority of the settlements while there are many tiles with few HSs.

### Density-independent classes of human settlements’ patterns

**Figure 2:**
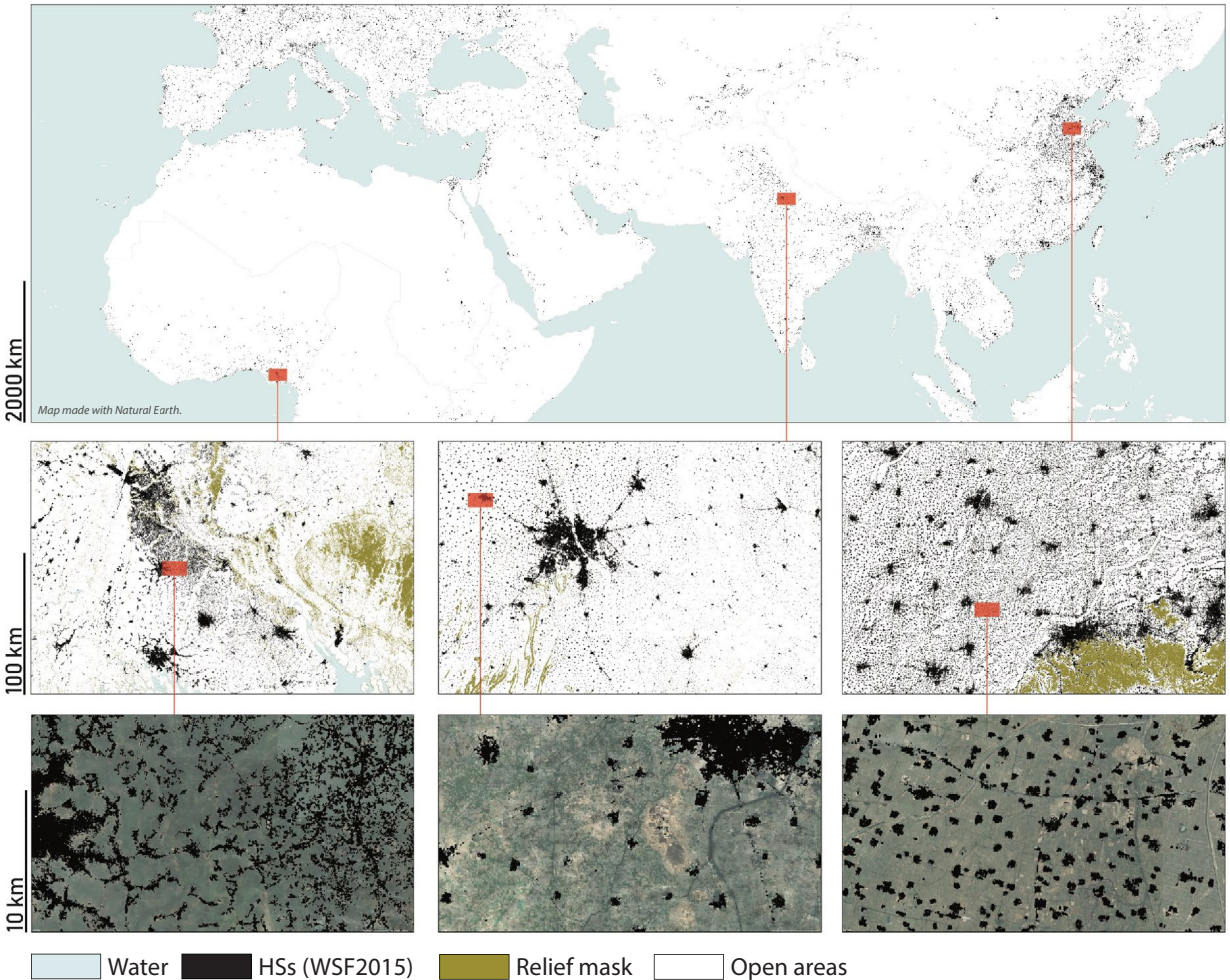
Three examples of the high heterogeneity of spatial patterns referring to the Igboland (Nigeria), New Delhi (India) and Jinan (China) regions. The HSs computed from the WSF2015 are shown in black, the relief mask in light brown, the water mask in blue, and the remaining open areas (including natural and cropland areas) in light green. In the bottom row, the background shows HR optical satellite imagery.

The spatial distribution of density alone does not explain the complexity of HSs patterns on Earth (see Fig. 2), which are very heterogeneous in shape and dimension. Such variety of patterns may arise from the very well-known spatial interpenetration of rural and urban settlements^37^, which results in a complexity of shapes and sizes that no longer fit those classes. This phenomenon has been qualitatively observed in classical urban geography narratives through the notions of *megalopolises*^37^, *urban sprawl*^38^ and *horizontal metropolises*^39^. However, this gradual symbiosis of different urbanization forces has never been quantitatively defined and tested. We here propose a quantitative classification of settlement patterns based on urban scaling^23,40^.

In the context of urbanization and HSs patterns analyses, some invariant spatial proprieties of HSs^23^ and transportation networks^41^ have been found to follow scale-free relationships. The strongest empirical evidence of a power-law relationship in urban science is the scale-free distribution of settlement sizes: the probability of observing a settlement with an area larger than *A* follows a power law, $P(A) \sim A^{-\alpha }$, also called Zipf’s law^31–34^. Accordingly, the areas of the HSs in the tile and those in its corresponding UN-defined macro-area *m* are expected to be sampled from the same empirical distribution, $P_{m}(A)$, which is well approximated by the Zipf’s law as expected (see SI, Fig. S1). Based on this assumption, for each HS *i* in a $0.5^{\circ } \times 0.5^{\circ }$ tile, we measure its area $A_{i}$ and the total HS area of a tile $A^{tot}_{HS} = \sum _{i=1}^N A_{i}$, where *N* is the number of HSs in the tile. Then, for each tile in macro-area *m* with a total settlement area $A^{tot}_{HS}$, we estimate $P_m(N|A^{tot}_{HS})$ following^42^. To do so, we randomly sample the HS areas from $P_{m}(A)$ until the sum of the sampled areas is equal to $A^{tot}_{HS}$ and find the number of HSs *N* we sampled. Then, we estimate the distribution $P(N | A^{tot}_{HS})$ by repeating the process 1000 times (see Methods). Note that the expected number of HSs increases with the total target area $A^{tot}_{HS}$. If the observed values of the number *N* of HSs is distributed according to the theoretical distribution $P_m(N | A^{tot}_{HS})$, then the corresponding quantiles $Q(N) = F(N | A^{tot}_{HS})$ should be distributed uniformly between 0 and 1, where *F* is the cumulative distribution of *N*.

**Figure 3:**
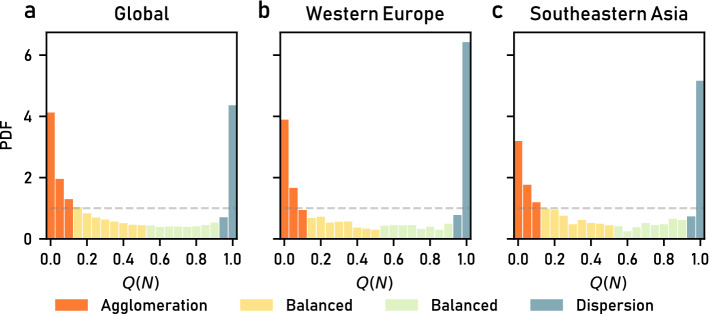
The distribution of the deviations from the Zipf’s law. Deviations with $Q(N)\simeq ~0$ correspond to tiles with a smaller number of HSs than expected, while when $Q(N)\simeq ~1$ tiles have a higher number of HSs than expected by the theoretical model. We observe two peaks in all the macroareas. (**a**) The global distribution; (**b**,**c**) two examples of distributions within a macroarea. The colors indicate our proposed classification.

However, we find that the empirical quantiles are not uniformly distributed between 0 and 1. Instead, we observe a bimodal distribution with two distinct peaks located around $Q(N)=0$ and $Q(N)=1$ (see Fig. 3). Similar results are observed in most macro-areas (see SI Fig. S2). Thus, based on the theoretical quantiles *Q*(*N*), we define two extreme classes of settlement patterns: a *dispersion class* ($0.9 \le Q(N) < 1$, 10th decile), corresponding to tiles with a large number of HSs according to the theoretical expectations; and an *agglomeration class* ($0.0 \le Q(N) < 0.1$, 1st decile), corresponding to tiles with a small number of HSs according to the theoretical expectations. In between these two extreme classes, we define the *balanced class* ($0.1 \le Q(N) < 0.9$, 2nd-9th deciles), divided into two sub-groups ($0.1 \le Q(N) < 0.5$ and $0.5 \le Q(N) < 0.9$) to better understand the patterns of the tiles.

**Figure 4:**
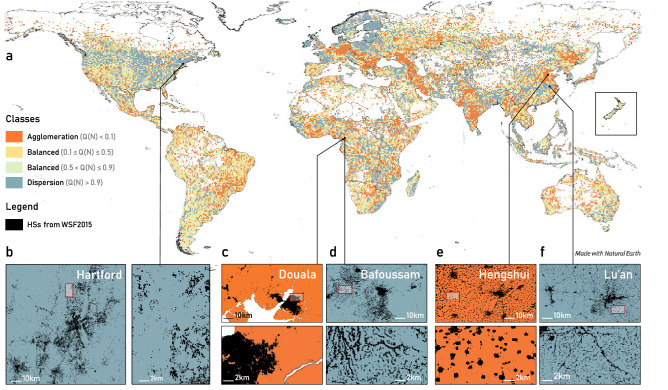
Global classification of the HS patterns by their deviation from the urban scaling predictions. The colour range is consistent across all panels. The blue insets (**b**,**d**,**f**) show the *dispersion* class, which represents locations with more HSs than expected from the scaling theory. (**b**) Two different zoom levels of Hartford’s metropolitan area (USA), where urban dispersion is due to extensive patterns of single housing and car-centric transportation. (**d**) Urban-rural agglomeration in the area around Bafoussam (Cameroon), where settlements are mostly composed of informal single housing/agricultural units. (**f**) Area around Lu’an (China), where there is an abundance of low-density sparse and small settlements. The orange insets (**c**,**e**) show the *agglomeration* class, in which we find fewer settlements than expected. (**c**) The city of Douala (Cameroon), where urbanization occurs tightly around the existing urban core. (**e**) The area around the city of Hengshui (China), where the many small settlements are most likely due to the agricultural land preservation strategies that have been extensively developed with a focus on compactness. In each 10 km pattern inset, we highlight with a red box the area showed by the 2 km zoom inset.

Figure 4a shows the spatial distribution of the classified tiles at a global scale. We observe that the tiles classes are not spatially distributed at random, but they tend to form spatially compact clusters. For example, the blue cluster in the dispersion class in southern China (in Fig. 4c) and the orange cluster in the *agglomeration* class in northern China (in Fig. 4e) are of considerable size and consist of multiple tiles. The fact that the classes of settlement patterns are not randomly distributed in space shows that the proposed classification scheme captures patterns characterizing large geographical regions and possibly large urban corridors. In North America, more precisely in the United States (US), we notice a large number of tiles in the dispersion class (blue tiles in Fig. 4a,b), whereas the rest of the tiles in the US are mostly within the *balanced* class (light yellow and green tiles), except for a few large urban agglomerations in the *agglomeration* class (orange tiles). This picture is in agreement with recent measurements of urban sprawl in US metropolitan areas and counties, which was evaluated using factors such as development density, land use mix, activity centring, and street accessibility^20^.

The proposed classification can highlight similarities and differences of HSs patterns observed on Earth. We find, for example, that highly compact cities, such as Douala, Cameroon (see Fig. 4c), belong to the same class of highly saturated areas like the city of Hengshui, China (see Fig. 4e). These areas may appear different at first glance; however, they are intrinsically similar, as in both cases, the settlements are compact, regardless of their spatial distribution. This classification is corroborated by a qualitative understanding of these two areas: Douala probably attracted all new settlers around the urban core as it is a port town and the wealthiest and most industrialized town in Cameroon, whereas, near Hengshui, the over-abundance of compactly developed settlements is due to avoidance of excessive erosion of productive agricultural land. By contrast, the Lu’an region (see Fig. 4f), which is also an agricultural area, belongs to the dispersion class probably because it has not been regulated by agricultural land erosion protection policies and thus presents a highly dispersed pattern of settlements. The same highly sprawled pattern appears in several mega-settlement agglomerations in sub-Saharan Africa, where large sub-urban areas are dominated by single-plot housing as in the area of Bafoussam, Cameroon (Fig. 4d).

We also observe that the observed bimodality in the deviations from scaling theory predictions cannot be explained by and is not a simple by-product of a different distribution of HS sizes for those tiles. The distributions of HS sizes for tiles in the dispersion class are indeed not systematically different from the distributions of the tiles in the *balanced* class (see SI, Fig. S3). The excess of tiles in the *dispersion* and agglomeration classes is observed across all values of the percentage of HS areas, $A^{tot}_{HS}$, indicating that an over-abundance of HSs is not specific to lowly or highly urbanized regions and is thus independent of urban density (see SI, Fig. S4).

### A spatial model for human settlements

The deviations from scaling theory predictions show a high heterogeneity of HSs patterns, resulting from numerous historical dynamics. In the absence of global and precise historical HSs data, we shed light on how such a variety might be achieved through controlled spatial simulations.

We hypothesize that HSs evolution cannot be attributed to agglomerating forces alone but rather to more complicated systems of spatial forces. To test this hypothesis, we here propose an extension of distance-weighted city growth models^34,43^ to simulate and reproduce such a system of forces and explain the macro-dynamics in action during settlement evolution. Our proposed model works in a two-dimensional lattice *w* of size $L \times L$, where $L=1000$, whose sites (cells) can be either occupied ($w_{i,j} = 1$, human settlement (HS)) or empty ($w_{i,j} = 0$, undeveloped). Without loss of generality, the initial configuration has only the central cell occupied ($w_{L/2, L/2} = 1$), and all other cells are empty ($w_{i, j} = 0\ \forall i,j \ne L/2$). Then, the model iteratively simulates the growth of settlements; at each step, the probability that each empty cell is occupied is:

$$\begin{aligned} q_{i,j} = C {\hat{q}}_{i,j} = C \frac{\sum _{k}^L\sum _{z}^L w_{k,z} d_{ij,kz}^{-\gamma }}{\sum _{k}^L \sum _{z}^L d_{ij,kz}^{-\gamma }} \end{aligned}$$

where $C = 1 / \max _{i,j}({\hat{q}}_{i,j})$ is a normalization constant and $d_{ij,kz}$ is the Euclidean distance between site $w_{i,j}$ and site $w_{k,z}$.

As in its traditional form^34^, the parameter $\gamma$ regulates the strength of attraction of an HS cell on a new cell; $\gamma = 0$ implies a dispersed and randomly located new occupied cell, while a larger $\gamma$ attracts new cells close to old cells, thus producing mono-centric and agglomerated patterns (see SI, Fig. S7). To simulate the different forces in action, the simulation is split in two steps that are controlled by the parameter *s*. When the fraction of the occupied cells in the simulation is less than a given percentage *s* (i.e., $\sum _{i,j} w_{i,j} / L^2 \le s$) $\gamma = \gamma _1$, while $\gamma = \gamma _2$ when the fraction of occupied cells is greater than *s*:

$$\begin{aligned} \gamma = {\left\{ \begin{array}{ll} \gamma _1, &{} \text {if}\ \sum _{i,j} w_{i,j} / L^2 \le s\\ \gamma _2, &{} \text {otherwise} \end{array}\right. } \end{aligned}$$

$\gamma _1$ characterises settlements’ expansion during the initial stages of the simulation while $\gamma _2$ characterises settlement expansion for the rest of the simulation (see SI, Fig. S8 for a visual explanation of the patterns generated). The model, which we call a multi-parameter model, has three parameters: $\gamma _1$, $\gamma _2$ and *s*. When $\gamma _1 = \gamma _2$, it becomes equivalent to the single-parameter model^34^.

**Figure 5:**
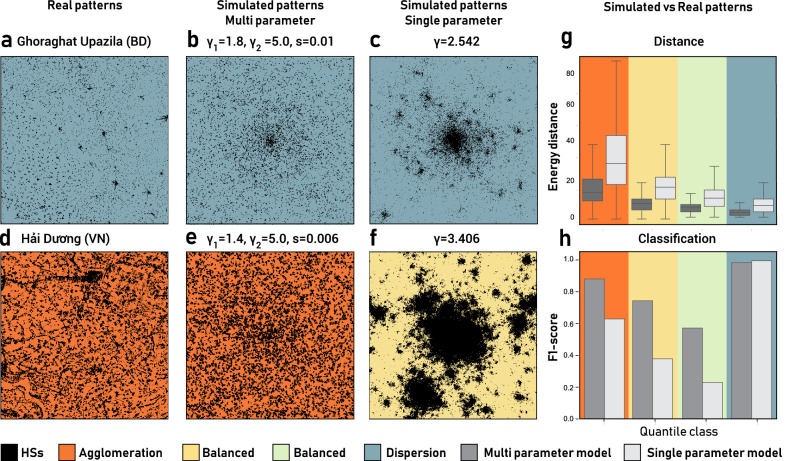
Qualitative and quantitative results of the proposed multi parameter model. (**a**) Area near Ghoraghat Upazila, Bangladesh, having a settlement pattern in the *dispersion* class; (**b**) the most similar simulation obtained with our model; (**c**) although the most similar simulation obtained with the single-parameter model lies in the same class as the real tile, it has a very different pattern. (**d**) Area near Hai Duong, Vietnam, having a settlement pattern in the *agglomeration* class; (**e**) the most similar simulation obtained with our model; (**f**) the most similar simulation obtained with the single-parameter model falls into the wrong class. (**g**) This box plot shows the energy distance between the real and simulated tiles computed for each class. It shows that our model always generates settlement patterns that are consistently better than those generated by the single-parameter model. (**h**) The F1-score obtained from the urbanization class of the real tile and the class of its most similar simulation for both our model and the single-parameter model. Our model outperforms the single-parameter model and generates settlement patterns compatible with the classes observed in the real world.

We follow a simulation approach in which we find the parameters that best represent the spatial process that might have generated the patterns of the real tiles. First, we generate approximately 1,000,000 simulations using a broad range of parameter values (see SI, Table S1) and simulate patterns until the lattice reaches 60% of occupied cells. For each real tile *i*, we find the most similar simulation by comparing the cumulative distributions of HS sizes and selecting the simulated tile with the smallest Wasserstein distance $D_{E}(i)$^44^ between the distribution of HS sizes of real and simulated tiles (see Methods). Finally, for each simulated tile, we also find its class of settlement patterns (i.e. *agglomeration*, *balanced*, *dispersion*) by the quantile procedure mentioned before. Figure 5a shows a randomly chosen tile in Ghoraghat Upazila, Bangladesh, while Fig. 5b shows its most similar simulation with parameters $\gamma _1 = 1.8$, $\gamma _2 = 5.0$ and $s=0.01$. This simulation describes the dispersal phase of the real tile well in both its HS pattern and the class of settlement patterns. The same cannot be said for the most similar simulation from the single-parameter model, as it fails to describe both the sprawled pattern (see Fig. 5c). Similarly, we see from Fig. 5d,e that the randomly chosen tile of Hai Duong, Vietnam, is very well described by our model with parameters $\gamma _1 = 1.4$, $\gamma _2 = 5.0$ and $s=0.006$, while the best simulation of the single-parameter model fails to simulate this large number of settlements in the *agglomeration* class and its class (Fig. 5f). More qualitative examples can be found in SI Figs. S9–S16.

To perform a quantitative evaluation of the performance of the multi-parameter model, we assess its ability to generate realistic distributions of HS sizes and urbanization classes. First, we compare the distributions of the Wasserstein distances $D_E$ across all the simulated tiles from the multi-parameter model and the single-parameter model (see Fig. 5g). The two-sided Kolmogorov-Smirnov (KS) test^45^ shows that the multi-parameter model has a significantly smaller distance for all urbanization classes (see SI, Table S1), with 45.85% smaller median distances, on average. This result is robust against different distance metrics (see SI for additional details). Second, we compare the urbanization class of each real tile with the one of its most similar simulation. We use the F1-score to quantify the agreement between the urbanization classes of the real and simulated tiles. We find that the multi-parameter model achieves 50.68% higher performance than that of the single-parameter model (see SI, Table S2). Fig. 5h shows that this increase in performance is evident for the *balanced* and *agglomeration* classes. We found that the single-parameter model overestimates the number of tiles in the *dispersion* class, while the multi-parameter model better captures the whole distribution of urbanization classes (see also SI Fig. S6). Moreover, we found out that the multi-parameter correctly simulates also the agglomeration-dispersion dichotomy we found in real data (see SI Fig. S17).

## Conclusion

Due to global population growth, HSs are expected to increase accordingly. For this reason, the scientific understanding of the spatial patterns of HSs is of paramount importance for planning, managing, and eventually forecasting HSs and their consequences.

In this paper, we provide an unprecedented description of the geography and the spatial structure of all HSs on Earth. First, we exploit the state of the global art dataset of human-occupied land to reliably measure the location and distribution of all the land occupied by HSs. We find that the density of HSs areas on Earth has a long-tail distribution: very few zones on Earth are occupied by highly dense areas, while the vast majority of Earth is occupied by low-density scattered settlements composed of less than $2\%$ of HSs area. These low-density and scattered patterns are not only the result of the expansion of metropolitan areas; they also depend on a different process that goes beyond the arbitrary rural-urban dichotomy. Cities are undoubtedly important to study for their socio-economic importance and *agglomeration* effects^46,47^. However, the long-tail distribution we find shows that the over-abundance of low-density areas occupy approximately 50% of the global surface, and may deserve more attention from the scientific community.

Second, we show that settlement density alone does not explain the great variability of HS patterns on a global scale. Thus, we exploit urban scaling findings to study the number of settlements expected to be found in a region with a given HS area. From the deviations of the urban scaling predictions, two distinct classes of settlement patterns emerge, which we named *dispersion* and *aggregation*. The former contains regions with the highest number of settlements with respect to their HS area, according to the deviations from urban scaling; conversely, the *agglomeration* class contains regions with the smallest number of settlements with respect to their HS area, according to the predictions of the urban scaling. We name the patterns between *aggregation* and *dispersion* as *balanced*. Recent seminal work has shown the tight relation between human settlements spatial distribution, $\hbox {CO}_2$ emissions and GDP^11,48,49^. Our global classification allows to understand and group the different patterns of HSs on Earth and thus might help better planning future policies for sustainable settlements’ growth.

Regarding the deviations from urban scaling, one can speculate that Zipf’s law is not fully capable of describing urban patterns. We instead argue that urban scaling is a valuable framework. We showed how deviations from Zipf’s law could be used to produce a quantitative classification of HS patterns, which provides additional insights to policy-makers and goes beyond the traditional rural-urban dichotomy.

Finally, we propose a spatially explicit model to shed light on the process that might result in the observed HSs patterns, in the absence of time-varying data at a global scale. The tiles we simulate match well with the HSs patterns and classifications, both locally (Fig. 4) and globally (see SI Fig. S6). The model is validated on multiple distance metrics and alternative baselines. Our findings show that the spatial dynamical process that regulates attractive and dispersal forces while settlements grow may be subject to random processes and that their combinations are undoubtedly subject to local and specific conditions. As such, local and regional conditions must be taken into account when studying and modelling urban phenomena.

A global and precise analysis of HSs does not come without limitations. It is worth noting that, due to limitations specific to the data used, it was not feasible to consistently and systematically detect globally tiny structures (e.g., huts, shacks, tents) due to their reduced scale, temporal nature (e.g., nomad or refugee camps), building material (e.g., cob, mud bricks, sod, straw, fabric), or the presence of dense vegetation preventing their identification. Moreover, we acknowledge that our simulations through the spatially explicit model find only a possible explanation for HSs’ observed patterns and classification on Earth. We stress the need for a global, precise and reliable time-varying dataset of HSs to better understand the spatial processes underlying HSs’ growth.

In our view, the analysis and model we propose represent a fundamental tool to provide insights about the structure and the evolution of HSs on Earth and, in turn, of their impact on humans and the environment. In the future, the observation of the Earth surface will experience tremendous improvement, providing more data that are more accurate and denser in time. We hope that our framework will pave the way to new research to understand the extent of HSs and manage better their impact on the environment and life on Earth.

## Methods

In this section we first describe how we delineate the HSs from satellite data, then we explain the relief mask and the segmentation process. Finally, we describe how we use the data to perform urban scaling, the simulations and the comparisons with the real data.

### Global HSs delineation from satellite imagery

We exploit the World Settlement Footprint 2015 dataset^35^ to reliably and accurately outline HSs globally. This dataset is composed of multiple binary raster files obtained from 2014-2015 multi-temporal Sentinel-1 radar and Landsat-8 optical imagery (of which approximately 107,000 and 217,000 scenes were processed, respectively). The dataset has an average resolution of 10 m, and it has been tested in close collaboration with Google for a collection of 50 globally distributed test sites (tiles of $1 \times 1$ lat/long degree), including 900,000 reference samples.

Physical environmental conditions play a significant role in HSs development; among these, terrain steepness is one of the most critical. Accordingly, to exclude from our analysis relief areas that are unfavourable for settlement, we generated - based on extensive empirical analysis - a binary mask using the Shuttle Radar Topography Mission (SRTM) Digital Elevation Model (DEM) available between -60$^\circ$ and +60$^\circ$ and the Advanced Spaceborne Thermal Emission and Reflection Radiometer (ASTER) DEM elsewhere. Specifically, the mask is positive where the shaded relief (depicting how the three-dimensional surface would be illuminated from a point light source) is greater than 212, or the roughness (defined as the largest inter-cell difference of a central pixel and its surrounding 8 cells) is greater than 15.

### Global vectorial HSs

Global urbanization is measured by taking into account HSs, water, and impervious areas. To facilitate the analysis at the global scale, the globe has been divided into a grid of $0.5 \times 0.5$ degrees in the European Petroleum Survey Group (EPSG) 4326 projection. Using a global water mask, we select only the cells that intersect the emerged lands, which results in 63,507 cells available for the analysis. First, we transform the raster databases into polygons at each cell through the GDAL 2.2.2  and PostGIS software packages. Next, we create a hierarchy of encapsulated grids where, at each level, a cell is composed of the four cells from the lower level (e.g., each cell of the $1 \times 1$ degree grid comprises four cells belonging to the $0.5 \times 0.5$ degree grid). At each level, the polygons are then merged on the boundaries of the lower level’s cells. The result is a series of layers where urbanization can be analyzed and processed worldwide at multiple scales.

The HSs, water and impervious areas are calculated in kilometres through the Universal Transverse Mercator (UTM) projections.

### Urban scaling

To numerically estimate the theoretical confidence intervals for the number of settlements *N* predicted by scaling theory, we proceed as follows. We evaluate the theoretical conditional distribution of the number of settlements in a tile of total HS area $A^{tot}_{HS}$, $P_m(N|A^{tot}_{HS})$, by sampling with replacement from the list of settlement areas belonging to the tile’s macro area *m* until the total HS area (i.e., the sum of the sampled areas) is equal to the target value $A^{tot}_{HS}$. The number of samples *N* needed to reach $A^{tot}_{HS}$ can be considered to be a number sampled from $P_m(N|A^{tot}_{HS})$. By repeating the sampling process 1000 times, we can evaluate the 1st and 9th deciles, corresponding to the boundaries of the *agglomeration* and *dispersion* classes, respectively.

### Evaluation of the multi-parameter model

Estimating the urbanization process would require temporal data, which are not easy to obtain. Moreover, a model fit on temporal data, where each pixel value is related to all the other pixels through a distance matrix, would be very computationally expensive. Indeed, each tile contains $n = 5567 \times 5567$ cells, and a full distance matrix would require ${\mathscr {O}}(n^2)$ memory. For this reason, we evaluate our model through simulations.

First, we simulate $1000 \times 1000$ tiles with an exhaustive grid search created from the Cartesian product of the “reasonable” values chosen for $\gamma _1, \gamma _2, s$ (see SI, Table S3). The set of all the simulation tiles is denoted by ${\mathscr {S}}$. Next, we compare the resulting simulations with the global (*real*) tiles. For each tile with an urbanization percentage $U_r \ge 1\%$, we find the simulated tile that is most similar to it by finding all the simulated tiles with an urbanization percentage $U_s \in [{U_r - 0.5\%}, {U_r + 0.5\%}]$. We compare the tiles via the Wasserstein distance $D(X_i, X_j)$, which is also known as Earth mover’s distance, between the distributions $X_i$ and $X_j$ of HSs areas in the real and simulated tiles, respectively. We denote the distance of a tile *i* to its most similar simulated tile by $D_E(i) = \min _{j \in {\mathscr {S}}} D(X_i, X_j)$. We also tested other distance measures but did not find significant differences (see SI Fig. S5). As the size of the simulations is $1000 \times 1000$ pixels, we resize the real tiles to the same dimension with a nearest-neighbour approach before applying the distance function. This resizing makes the tiles comparable.

For each pair (*r*, *s*), where *r* is the real $1000 \times 1000$ pixel tile and *s* is the simulated $1000 \times 1000$ pixel tile, we compute the quantile class of *r* and *s* and frame it as a classification problem. We compute the F1-score between the ground truth (the classes of the real tiles) and the predicted classes (the classes of the simulated tiles). The F1-score for all the classes is weighted to account for the unbalanced number of tiles in each class.

## Supplementary Information

Supplementary Information.

## Data Availability

This research is based upon data openly available in the Internet. We release the code and the instructions to download all the source and intermediate data to repeat all the analysis and replicate the figures at https://github.com/denadai2/precise-mapping-human-settlements.

## References

[1] Leung B (2020). Clustered versus catastrophic global vertebrate declines. Nature.

[2] UN. *Cities in a Globalizing World: Global Report on Human Settlements 2001* (Earthscan, 2001).

[3] UN. *The State of the World Cities 2004/5: Globalization and Urban Culture.* (Routledge, 2004).

[4] UN. *The State of the World Cities 2006/7: The Millennium Development Goals and Urban Sustainability.* (Routledge, 2006).

[5] Birch EL, Wachter SM (2011). Global Urbanization.

[6] Moore M, Gould P, Keary BS (2003). Global urbanization and impact on health. Int. J. Hyg. Environ. Health.

[7] Zhou L (2004). Evidence for a significant urbanization effect on climate in china. Proc. Natl. Acad. Sci. USA.

[8] Kaufmann RK (2007). Climate response to rapid urban growth: Evidence of a human-induced precipitation deficit. J. Clim..

[9] Grimm NB (2008). Global change and the ecology of cities. Science.

[10] Tilman D, Balzer C, Hill J, Befort BL (2011). Global food demand and the sustainable intensification of agriculture. Proc. Natl. Acad. Sci. USA.

[11] Ribeiro HV, Rybski D, Kropp JP (2019). Effects of changing population or density on urban carbon dioxide emissions. Nat. Commun..

[12] Daily GC, Ehrlich PR (1992). Population, sustainability, and earth’s carrying capacity. BioScience.

[13] Johnson MP (2001). Environmental impacts of urban sprawl: A survey of the literature and proposed research agenda. Environ. Plan. A.

[14] Dye C (2008). Health and urban living. Science.

[15] Seto KC, Fragkias M, Güneralp B, Reilly MK (2011). A meta-analysis of global urban land expansion. PLoS ONE.

[16] d’Amour CB (2017). Future urban land expansion and implications for global croplands. Proc. Natl. Acad. Sci. USA.

[17] Güneralp B (2017). Global scenarios of urban density and its impacts on building energy use through 2050. Proc. Natl. Acad. Sci. USA.

[18] Herold M, Scepan J, Clarke KC (2002). The use of remote sensing and landscape metrics to describe structures and changes in urban land uses. Environ. Plan. A.

[19] Barrington-Leigh C, Millard-Ball A (2015). A century of sprawl in the United States. Proc. Natl. Acad. Sci. USA.

[20] Hamidi S, Ewing R (2014). A longitudinal study of changes in urban sprawl between 2000 and 2010 in the United States. Landsc. Urban Plan..

[21] Huang J, Lu XX, Sellers JM (2007). A global comparative analysis of urban form: Applying spatial metrics and remote sensing. Landsc. Urban Plan..

[22] Poelmans L, Van Rompaey A (2009). Detecting and modelling spatial patterns of urban sprawl in highly fragmented areas: A case study in the Flanders–Brussels region. Landsc. Urban Plan..

[23] Batty M (2008). The size, scale, and shape of cities. Science.

[24] Potere D, Schneider A (2007). A critical look at representations of urban areas in global maps. GeoJournal.

[25] Gamba P, Herold M (2009). Global Mapping of Human Settlement e Experiences, Datasets, and Prospects.

[26] Grekousis G, Mountrakis G, Kavouras M (2015). An overview of 21 global and 43 regional land-cover mapping products. Int. J. Remote Sens..

[27] Angel S, Parent J, Civco DL, Blei A, Potere D (2011). The dimensions of global urban expansion: Estimates and projections for all countries, 2000–2050. Prog. Plan..

[28] Pesaresi, M. *et al.**Operating Procedure for the Production of the Global Human Settlement Layer from Landsat Data of the Epochs 1975, 1990, 2000, and 2014* (Tech. Rep, European Join Research Center, 2016).

[29] Chen J (2017). 30-meter global land cover data product-globe land30. Geomatics World.

[30] Esch T (2017). Breaking new ground in mapping human settlements from space-the global urban footprint. ISPRS J. Photogramm. Remote Sens..

[31] GK, Z. *Human Behavior and the Principle of Least Effort.* (Addison-Wesley, 1949).

[32] Rozenfeld HD (2008). Laws of population growth. Proc. Natl. Acad. Sci. USA.

[33] Gabaix X, Ioannides YM (2004). The evolution of city size distributions. Handb. Region. Urban Econ..

[34] Rybski D, Ros AGC, Kropp JP (2013). Distance-weighted city growth. Phys. Rev. E.

[35] Marconcini M (2020). Outlining where humans live, the world settlement footprint 2015. Sci. Data.

[36] Office, U. N. S. *Standard Country or Area Codes for Statistical Use*, vol. 42 (UN, 1982).

[37] Gottmann J (1957). Megalopolis or the urbanization of the northeastern seaboard. Econ. Geogr..

[38] Indovina F, Matassoni F, Savino M. La (1990). città diffusa.

[39] Viganò, P., Arnsperger, C., Lanza, E. C., Corte, M. B. & Cavalieri, C. Rethinking urban form: Switzerland as a “horizontal metropolis”. *Urban Plan.* **2**, 88 (2017).

[40] Auerbach F (1913). Das gesetz der bevölkerungskonzentration. Petermanns Geogr. Mitteilungen.

[41] Strano E, Nicosia V, Latora V, Porta S, Barthélemy M (2012). Elementary processes governing the evolution of road networks. Sci. Rep..

[42] Simini F, James C (2019). Testing heaps’ law for cities using administrative and gridded population data sets. EPJ Data Sci..

[43] Makse HA, Andrade JS, Batty M, Havlin S, Stanley HE (1998). Modeling urban growth patterns with correlated percolation. Phys. Rev. E.

[44] Vaserstein LN (1969). Markov processes over denumerable products of spaces, describing large systems of automata. Probl. Peredachi Inf..

[45] Kendall MG (1938). A new measure of rank correlation. Biometrika.

[46] Bettencourt LM, Lobo J, Helbing D, Kühnert C, West GB (2007). Growth, innovation, scaling, and the pace of life in cities. Proc. Natl. Acad. Sci. USA.

[47] Gomez-Lievano A, Patterson-Lomba O, Hausmann R (2016). Explaining the prevalence, scaling and variance of urban phenomena. Nat. Hum. Behav..

[48] Ribeiro HV, Oehlers M, Moreno-Monroy AI, Kropp JP, Rybski D (2021). Association between population distribution and urban GDP scaling. PLoS ONE.

[49] Zhou B (2020). A gini approach to spatial Co2 emissions. PLoS ONE.

