# Supplementary material for: The agglomeration and dispersion dichotomy of human settlements on Earth
Source: Sci Rep. 2021 Dec 2;11:23289. doi: 10.1038/s41598-021-02743-9 (PMC8640067; doi:10.1038/s41598-021-02743-9)
Supplement: Supplementary file 1 — Supplementary Information. [file 41598_2021_2743_MOESM1_ESM.pdf]

The agglomeration and dispersion dichotomy of human settlements on Earth

Emanuele Strano*,[1] Filippo Simini*,[2] Marco De Nadai*,[3] Thomas Esch,[4] and Mattia Marconcini[4]

[1] *MindEarth, 2502 Biel/Bienne, CH*

[2] *University of Bristol, 06010 Bristol, UK*

[3] *Fondazione Bruno Kessler (FBK), 38123 Trento, Italy*

[4] *German Aerospace Center (DLR), 82234 Wessling, Germany*

ADDITIONAL FIGURES

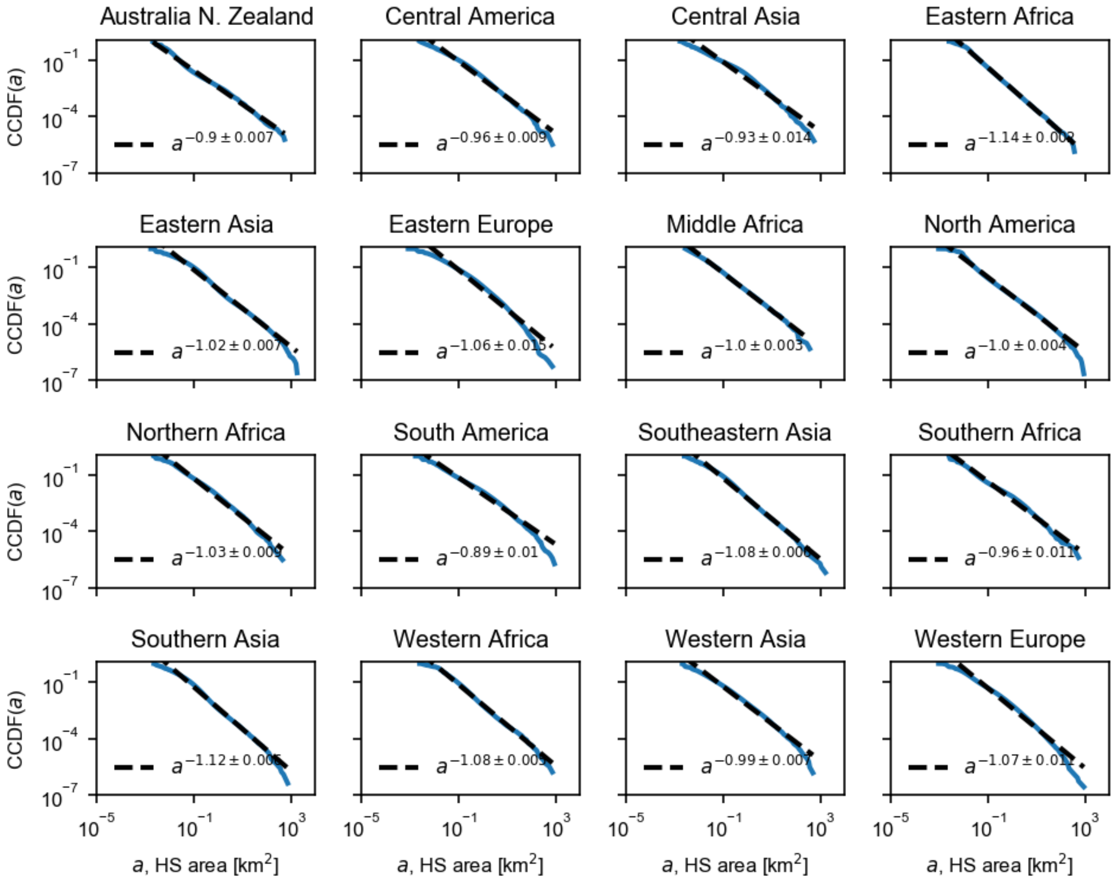

Figure S1. Counter cumulative distribution function (CCDF) of the HS areas for the 16 macro regions considered.

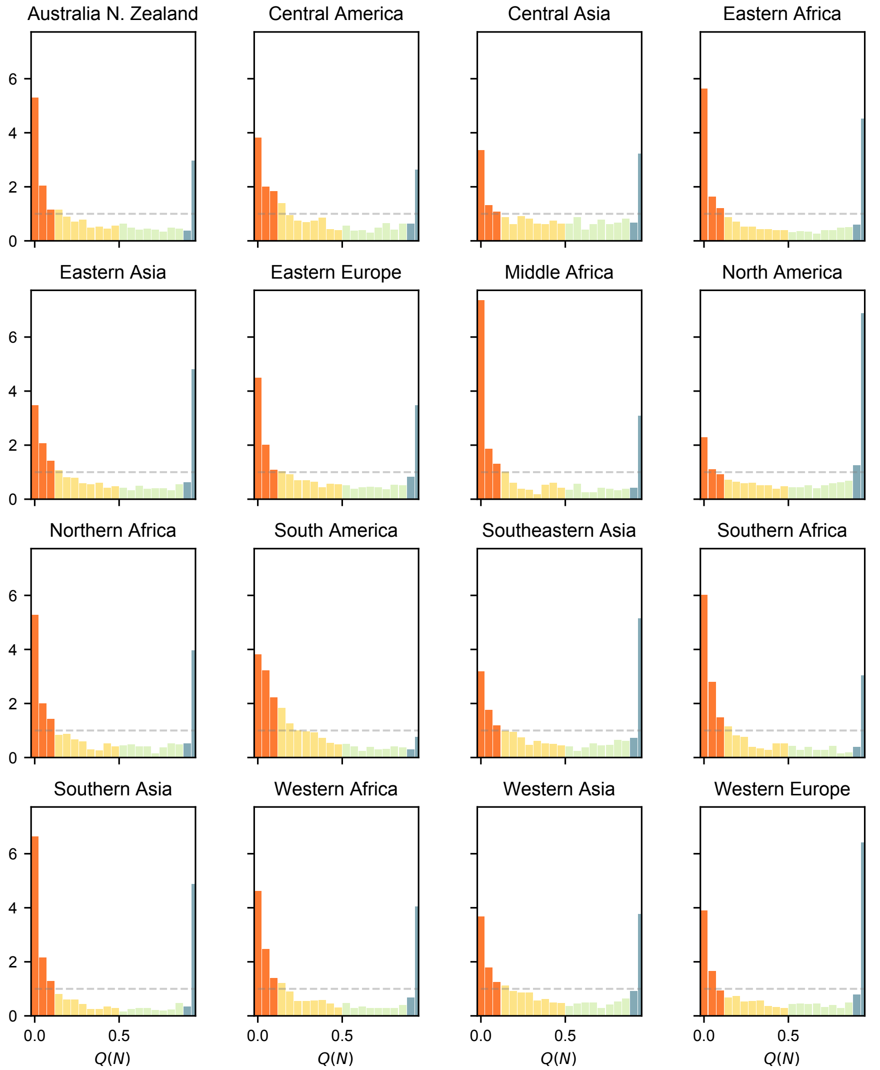

Figure S2. Quantile of the number of BUCs in a tile according to the theoretical distribution $P(N|A_{BUC}^{tot})$.

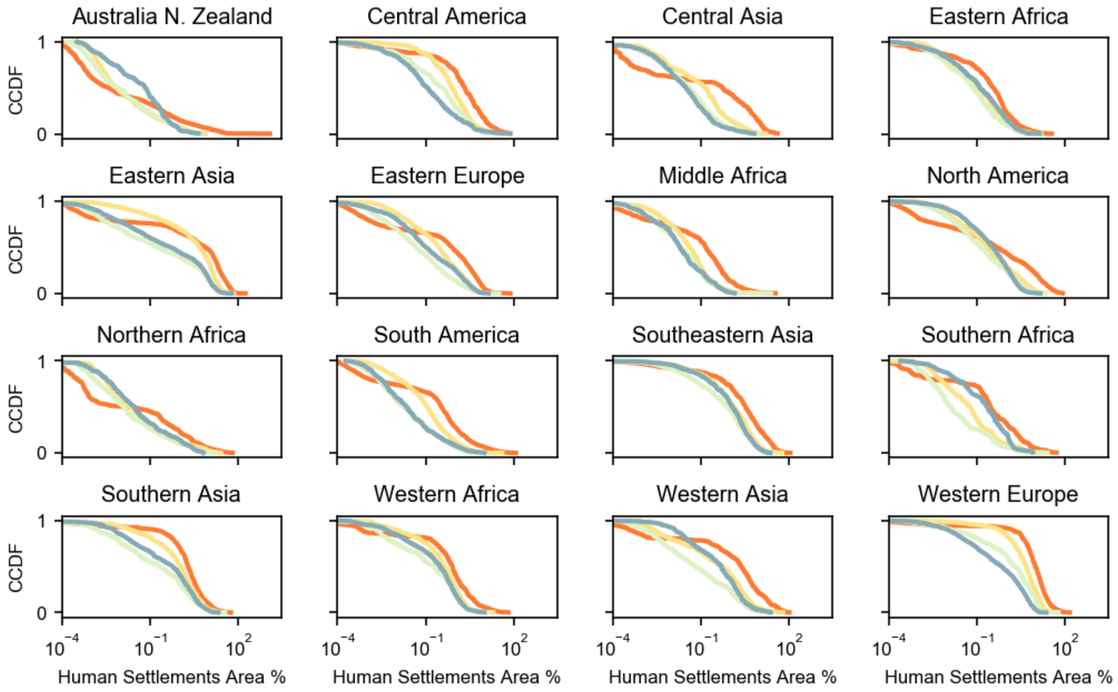

Figure S3. Counter cumulative distribution function (CCDF) of the HS areas separately for each class of settlement patterns: Dispersion (blue), Balanced (green and yellow) and Agglomeration (orange). Not surprisingly, the tiles in the Agglomeration class contain a higher number of large clusters. The cluster size distributions of the tiles in the Dispersion class are not consistently different from those in the Balanced class.

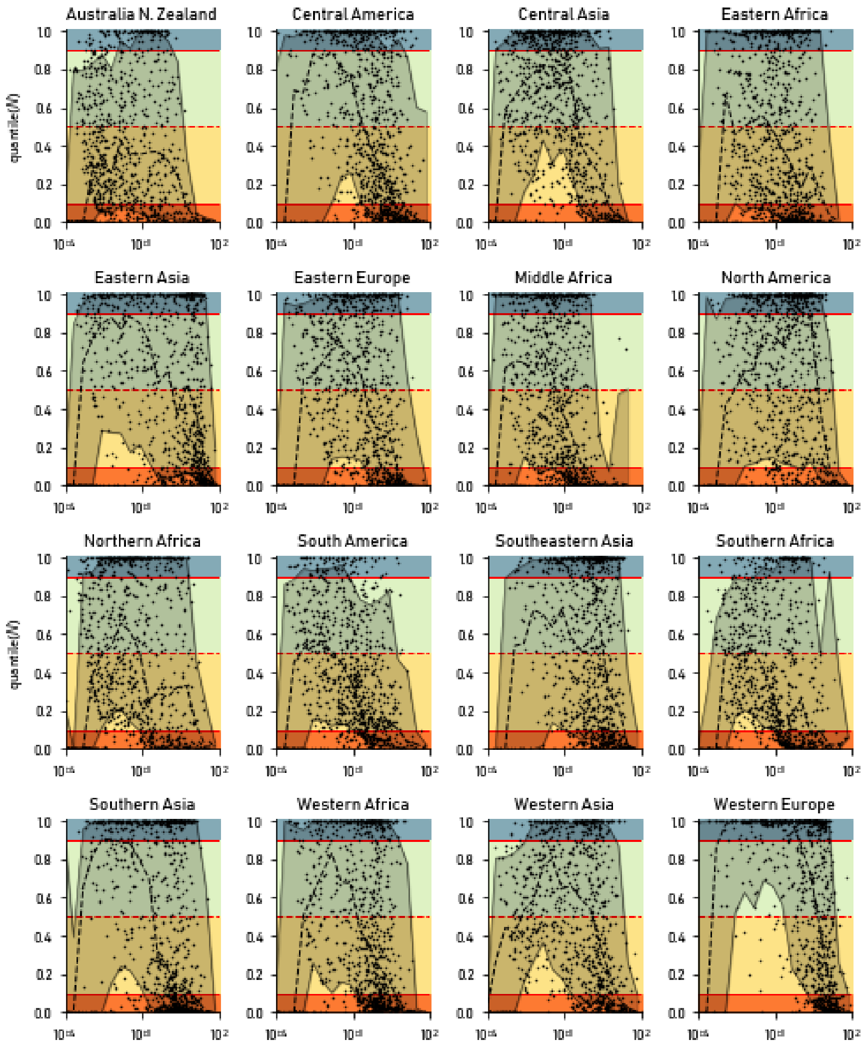

Figure S4. Scatter distribution of the quantile of the number of BUCs in a tile according to the theoretical distribution $P(N|A_{BUC}^{tot})$.

ROBUSTNESS TESTS

We run several tests to verify the sensitivity of the predictive model to:

- *Alternative metrics for the matches.* The match between real and simulated tiles might be sensitive to the choice of the similarity metric. Therefore, in Figure S5, we show the results with the energy distance [?] (i.e. Wasserstein-2 distance), the Jensen-Shannon (JS) divergence, and the Wasserstein distance (i.e. Earth mover's distance). Table S1 shows the same result broken down per class. The energy distance is defined as the distance between two independent random variables X, Y as:

$$D^2(X,Y) = 2\mathbb{E}\left|X-Y\right| - \mathbb{E}\left|X-X'\right| - \mathbb{E}\left|Y-Y'\right| \tag{S1}$$

where $\mathbb{E}\left|X\right| < \infty$, $\mathbb{E}\left|Y\right| < \infty$, X' is an iid copy of X and Y' is an iid copy of Y. The JS divergence is instead defined as:

$$D_{\mathrm{JS}}(P \parallel Q) = \frac{1}{2}D_{\mathrm{KL}}(P \parallel M) + \frac{1}{2}D_{\mathrm{KL}}(Q \parallel M)$$

where P and Q are discrete probability and $M = \frac{1}{2}(P+Q)$.

- *The multi-prob parameter model.* We also test for a different formalization of the multi-parameter model where the exponent γ is not changed only one time but is instead chosen at random with a specified probability in each stage of the simulation process. We simulate the growth in urban area through a two-dimensional $N \times N$ lattice whose sites $w_{i,j}$ can be either occupied ($w_{i,j} = 1$) or empty ($w_{i,j} = 0$). Without loss of generality, we set the initial configuration with $w_{N/2,N/2} = 1$ and all other pixels are zeros. Then, we simulate an evolution process where in each step, the probability that each empty site will be occupied is:

$$q_{i,j} = C\frac{\sum_k^N \sum_z^N w_{k,z} d_{k,z}^{-\Gamma}}{\sum_k^N \sum_z^N d_{k,z}^{-\Gamma}}$$

where $C = 1/max_{i,j}(q_{i,j})$ is a normalization constant for each step and $d_{k,z}$ is the Euclidean distance between site $w_{i,j}$ and site $w_{k,z}$. Γ is chosen based on a number p that is randomly chosen in each step:

$$\Gamma = \begin{cases} \gamma_1, & \text{if } p < s \\ \gamma_2, & \text{otherwise} \end{cases}$$

where s is a chosen probability threshold of the simulation and γ_1, γ_2 are selected growth parameters of the simulation. In each step, $w_{i,j} = 1$ iff $q_{i,j} > 0.5$. We stop the growth of

urban areas when $\frac{1}{N^2}\sum_{i,j}^{N} w_{i,j} \geq 0.5$. Since we choose the Γ parameter in each step, we call this the multi-prob parameter model, whereas the other one is called multi-parameter model. Table S3 shows the simulated parameters. Table S2 shows that the alternative formulation has comparable results of the presented mode, in terms of the F1-score between classes.

Together, these results confirm the robustness of our models and methods.

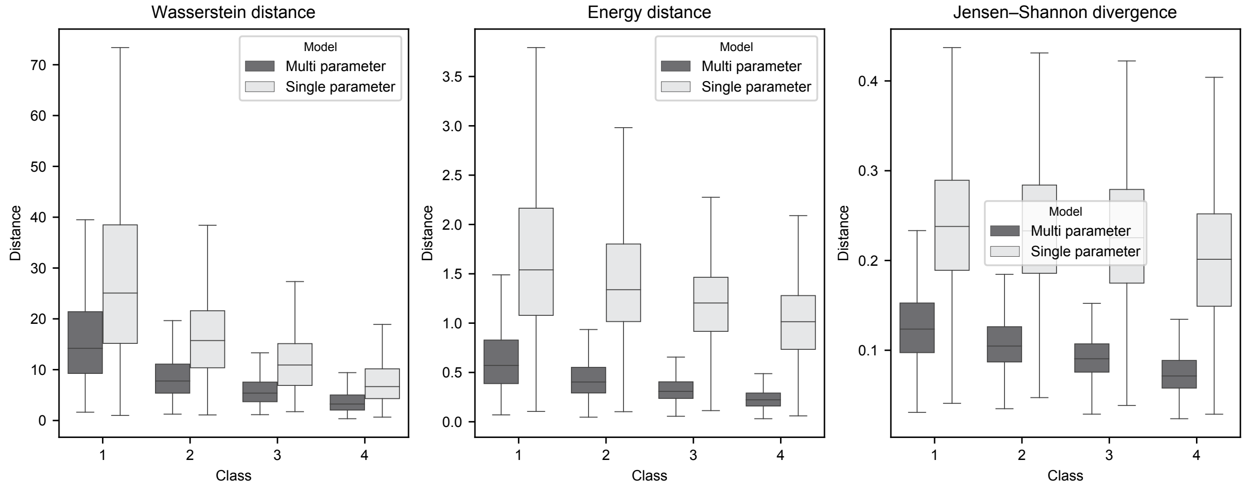

Figure S5. Distance between each real tile and its best simulation for the multi-parameter and single-parameter models. Our model consistently achieves shorter distances between simulated and real tiles, even when using alternative metrics such as the Energy distance and the Jensen–Shannon divergence.

Table S1. 2-way Kolmogorov-Smirnov test of the empirical distribution of the distances between real tiles and simulated tiles for the various models and the single-parameter model. The multi parameter model we propose achieve better performance than the single-parameter model in all classes. (**) indicates a p-value < 0.001.

**Method**	**Agglomeration**	**Balanced**		**Dispersion**	**All**
**Multi parameter**	0.34**	0.47**	0.48**	0.41**	0.29**
Multi-prob parameter	0.29**	0.38**	0.39**	0.37**	0.26**

Table S2. F1-score between the urbanization class of the real tile and the urbanization class of its most similar simulation tile. The multi-parameter model achieves the best performance in all classes.

**Method**	**Agglomeration**	**Balanced**		**Dispersion**	**All**
Single parameter	0.66	0.39	0.24	0.99	0.48
**Multi parameter**	0.88	0.74	0.56	0.98	0.73
Multi-prob parameter	0.89	0.76	0.61	0.91	0.74

SIMULATED PARAMETERS

In Table S3 we show all the tested parameters (and their combinations) for the Multi-parameter and the Multi-prob parameter models.

Table S3. Parameters used to perform the simulations. The simulations are created from the Cartesian product of these parameters. In the multi-prob parameter model for $s = 0.5$, we computed only those combinations where $y_1 < y_2$, as the probability to choose one gamma is 0.5.

**Parameters**	**Values**
**Single parameter model**	
γ_1	all parameters with 0.002 step from 1 to 10
**Multi parameter model**	
γ_1	$\{1, 1.4, 1.8, 2, 2.2, 2.4, 2.6, 2.8, 3, 3.2, 3.4, 3.6, 3.8, 4, 5, 6, 7, 8, 10\}$
γ_2	$\{1, 1.4, 1.8, 2, 2.2, 2.4, 2.6, 2.8, 3, 3.2, 3.4, 3.6, 3.8, 4, 5, 6, 7, 8, 10\}$
s	$\{.0002, .00005, .0008, .0001, .0004, .0006,$ $.001, .002, .004, .006, .008, .01, .02, .03, .04, .05, .06, .07, .08, .09,$ $.1, .2, .3, .4, .5\}$
**Multi-prob parameter model**	
γ_1	$\{1, 1.4, 1.8, 2, 2.2, 2.4, 2.6, 2.8, 3, 3.2, 3.4, 3.6, 3.8, 4, 5, 6, 7, 8, 10\}$
γ_2	$\{1, 1.4, 1.8, 2, 2.2, 2.4, 2.6, 2.8, 3, 3.2, 3.4, 3.6, 3.8, 4, 5, 6, 7, 8, 10\}$
s	$\{0.5, 0.51, 0.52, 0.54, 0.57, 0.59, 0.61,$ $0.64, 0.66, 0.68, 0.71, 0.73, 0.75, 0.77, 0.79, 0.82, 0.84, 0.86, 0.89,$ $0.91, 0.93, 0.96, 0.98, 0.99\}$

GLOBAL HSS DENSITY

In Table S4 we show the exact number of the density of HSs in all the macro-areas.

Table S4. Cumulative probability of HS areas for all the areas around the globe.

**Method**	**Lower bounds of the bins**					
Australia N. Zealand	0.761	0.978	0.991	0.995	0.998	0.999
Eastern Africa	0.246	0.910	0.980	0.996	0.999	1.000
Middle Africa	0.481	0.983	0.994	0.998	0.999	0.999
Northern Africa	0.739	0.958	0.982	0.996	0.999	0.999
Southern Africa	0.386	0.911	0.964	0.993	0.998	0.999
Western Africa	0.415	0.873	0.963	0.991	0.999	0.999
Central America	0.145	0.732	0.890	0.966	0.995	0.999
North America	0.491	0.855	0.937	0.975	0.995	0.999
South America	0.453	0.946	0.982	0.994	0.998	0.999
Central Asia	0.537	0.928	0.961	0.986	0.998	0.999
Eastern Asia	0.454	0.673	0.741	0.832	0.970	0.995
Southeastern Asia	0.120	0.540	0.752	0.910	0.988	0.998
Southern Asia	0.156	0.550	0.827	0.963	0.998	0.999
Western Asia	0.427	0.820	0.936	0.979	0.997	0.999
Eastern Europe	0.577	0.857	0.941	0.991	0.999	0.999
Western Europe	0.095	0.385	0.572	0.833	0.990	0.999

GLOBAL CLASSIFICATION OF THE HSS PATTERNS

In Figure S6 we show the classification of all the tiles with more than 1% urbanization. In this figure we compare the real tiles with the simulated ones.

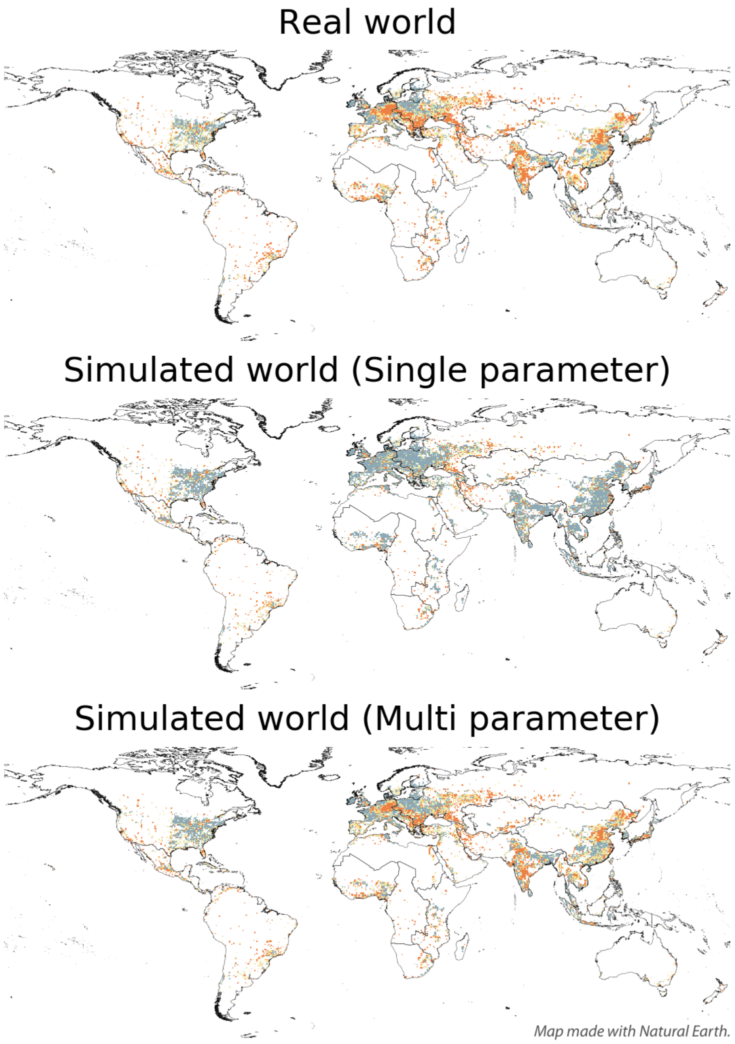

Figure S6. Global classification of all the tiles with more than 1% urbanization for the real tiles (first row) and the simulated ones with the Single-parameter model (second row) and the Multi-parameter model (third row). As it can be seen, the Single-parameter model overestimates the number of tiles in the Dispersion and Agglomeration classes. The multi-parameter model mitigates this problem and it is very similar to the real classification of the tiles (as shown by the F1-score accuracy over the different classes). This means that the Multi-parameter model reliably simulates the global pattern of urbanization. Made with Natural Earth.

SOME EXAMPLES OF SIMULATIONS

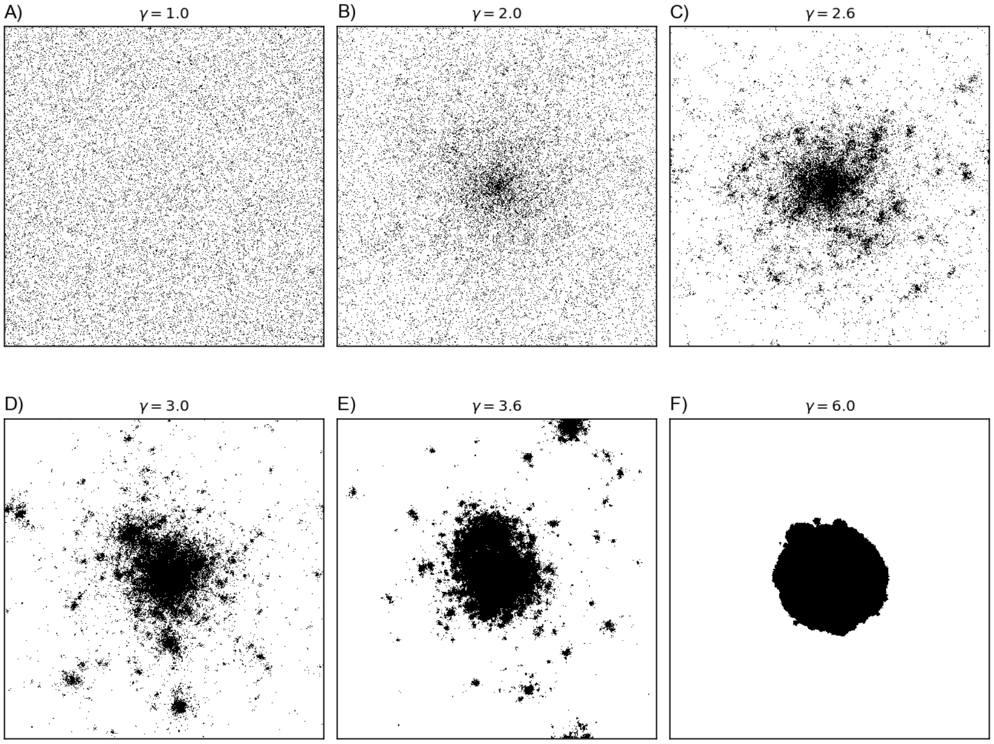

Figure S7. Different simulations of the single-parameter model with 30% of total BUC at different γ values. Low values of γ generate dispersed settlement patterns, whereas high values of γ generate compact patterns. It can be seen that in A) the model generates a random noise pattern, as the urban areas are created without caring on the existing urban areas ($\gamma = 0.1$). Contrarily, in F) a dense urban pattern is generated, as the model creates new urban ares with high probability only near those areas that are already built-up.

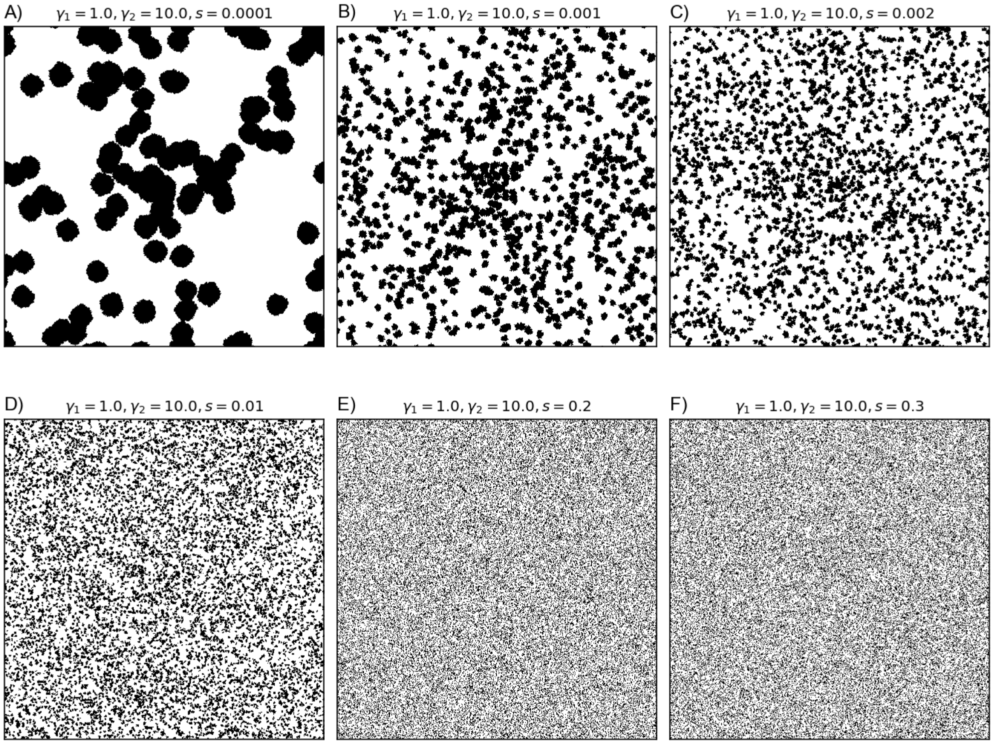

Figure S8. Different simulations of the Multi-parameter model with 30% of total BUC for the same γ_1 and γ_2 but different s values. It can be seen that in A) the model starts with a sparse pattern ($\gamma_1 = 0.1$) and then switches to the dense one ($\gamma_1 = 10.0$) until 30% of urbanization. The resulting pattern is clustered in circles. Contrarily, in F) a random noise pattern is created, as the urban areas are created without caring on the existing urban areas ($\gamma_1 = 0.1$). The parameter $\gamma_1 = 0.1$ is kept until the end of the simulation. The Multi-parameter model generates more complex patterns than the Single-parameter model.

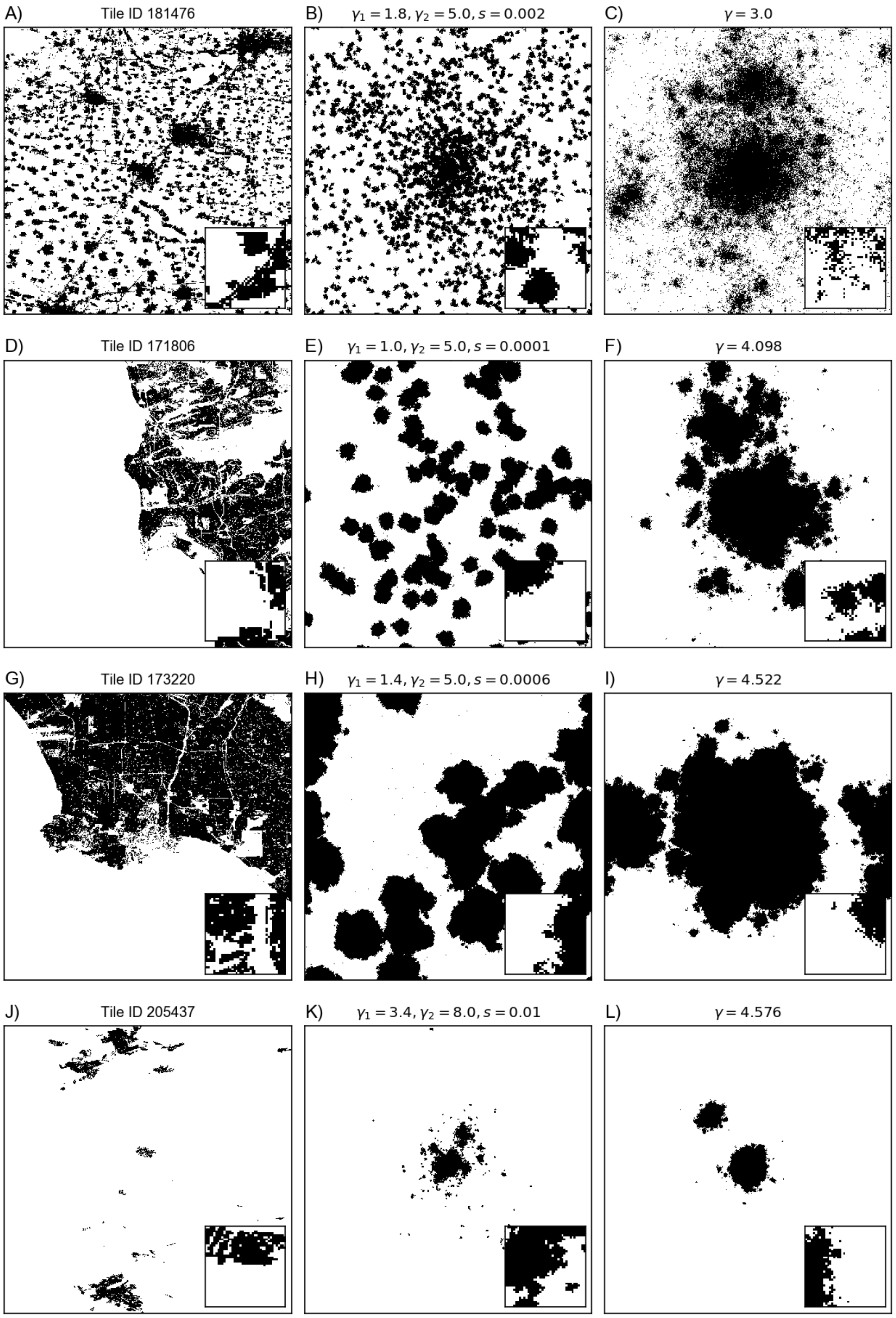

Figure S9. Some examples of the best simulations for four tiles in the Agglomeration class. The left column shows the real tile, the central column shows the most similar tile generated with the multi-parameter model, and the right column shows the single-parameter model simulation that is most similar to the real tile.

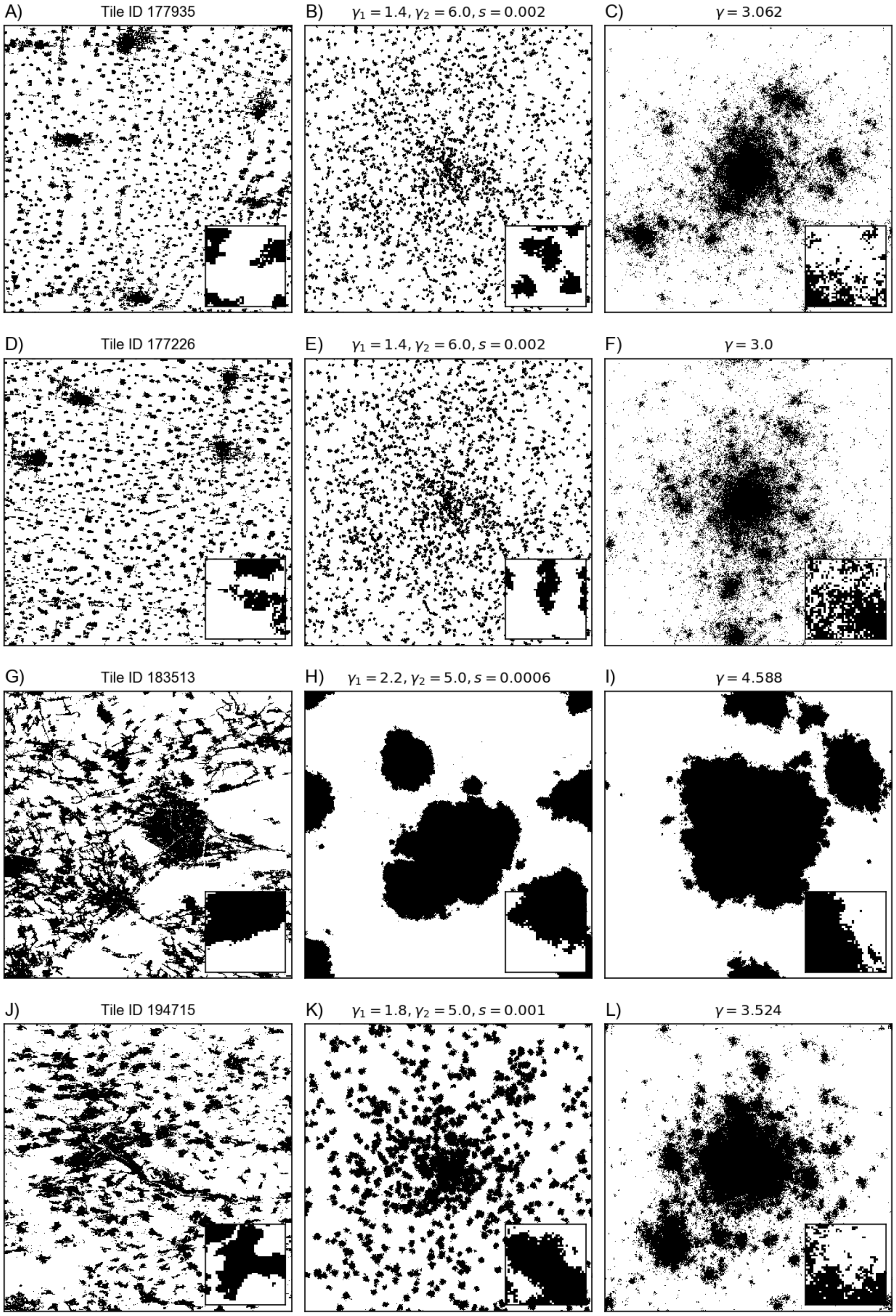

Figure S10. Some more examples of the best simulations for four tiles in the Agglomeration class. The left column shows the real tile, the central column shows the most similar tile generated with the multi-parameter model, and the right column shows the single-parameter model simulation that is most similar to the real tile.

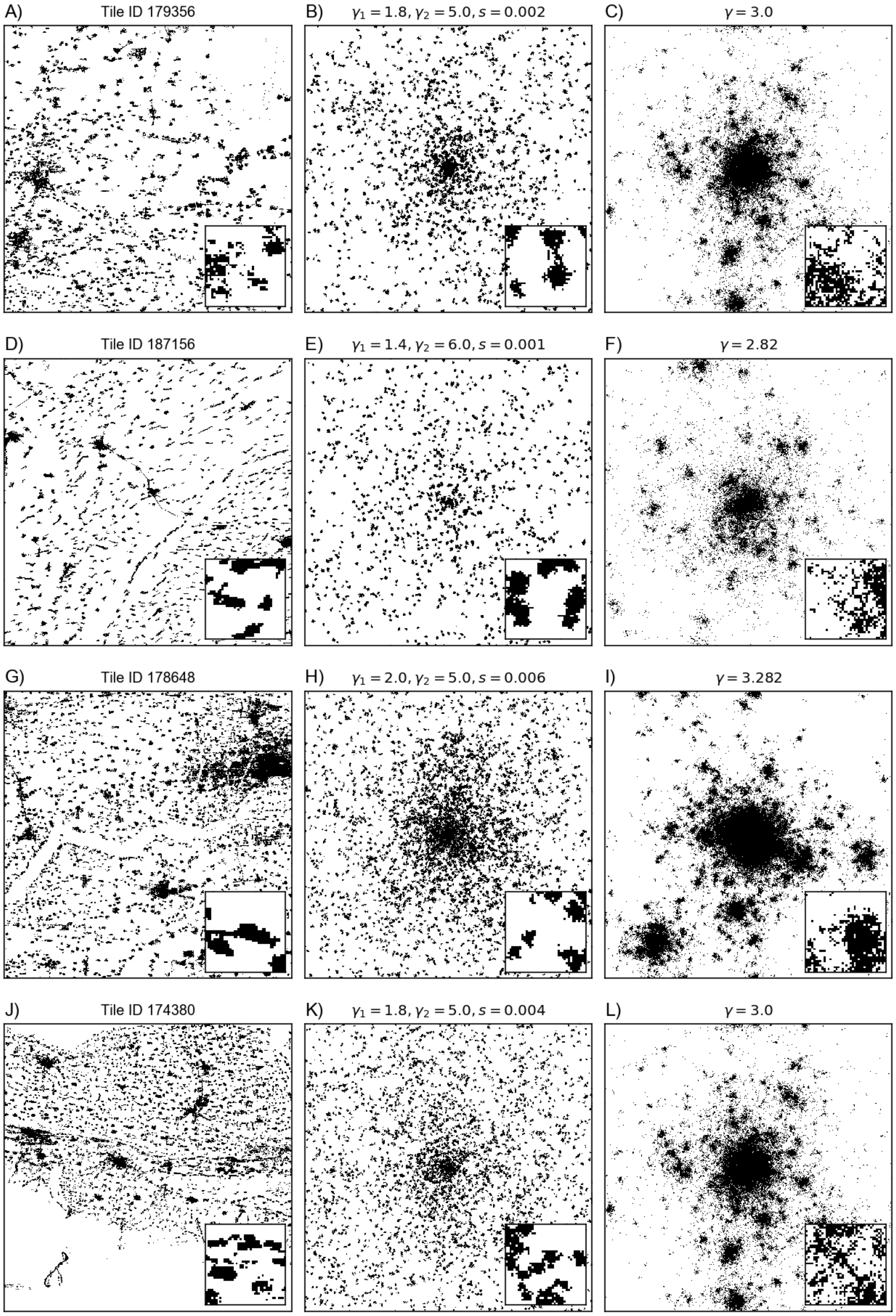

Figure S11. Some examples of the best simulations for four tiles in the Balanced class (yellow group). The left column shows the real tile, the central column shows the most similar tile generated with the multi-parameter model, and the right column shows the single-parameter model simulation that is most similar to the real tile.

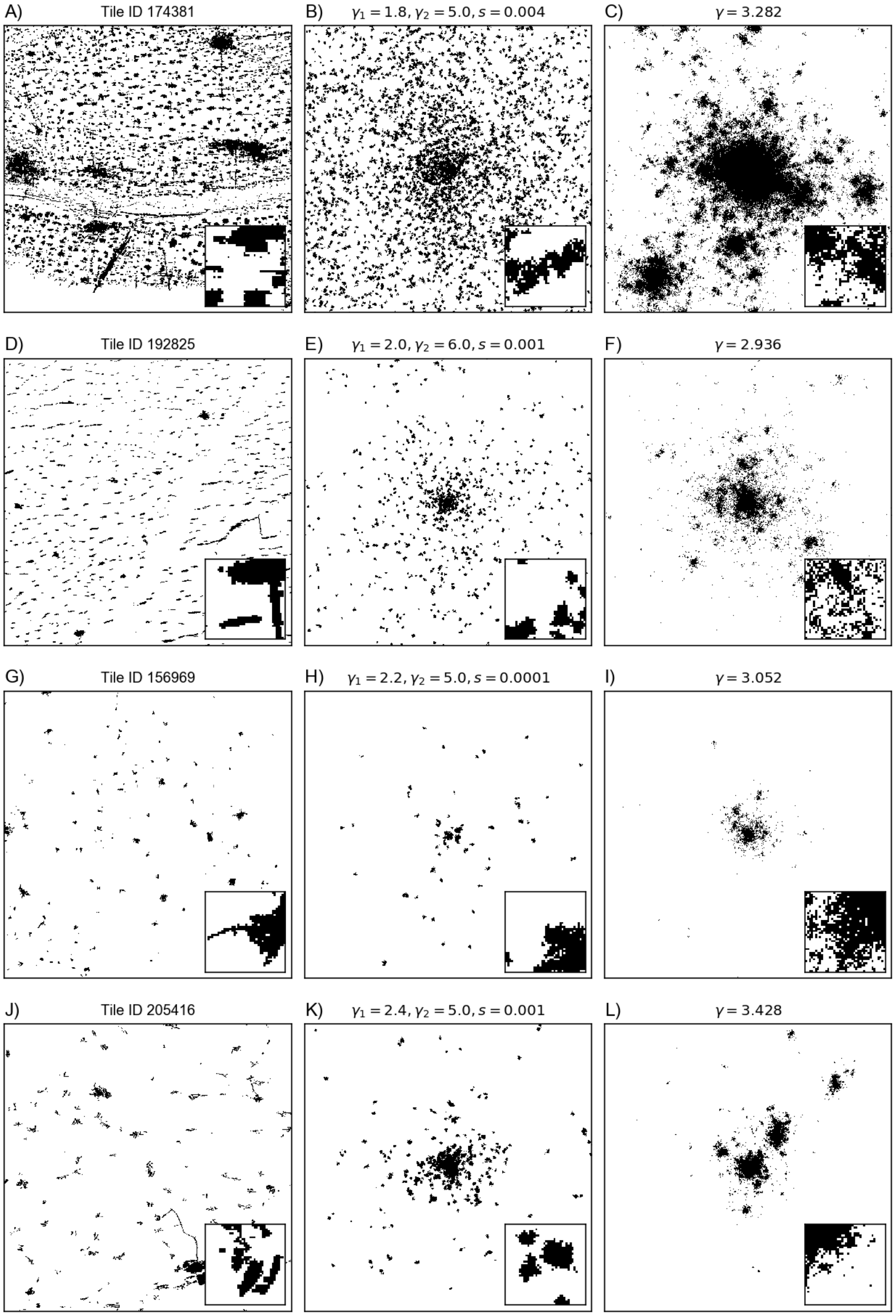

Figure S12. Some more examples of the best simulations for four tiles in the Balanced class (yellow group). The left column shows the real tile, the central column shows the most similar tile generated with the multi-parameter model, and the right column shows the single-parameter model simulation that is most similar to the real tile.

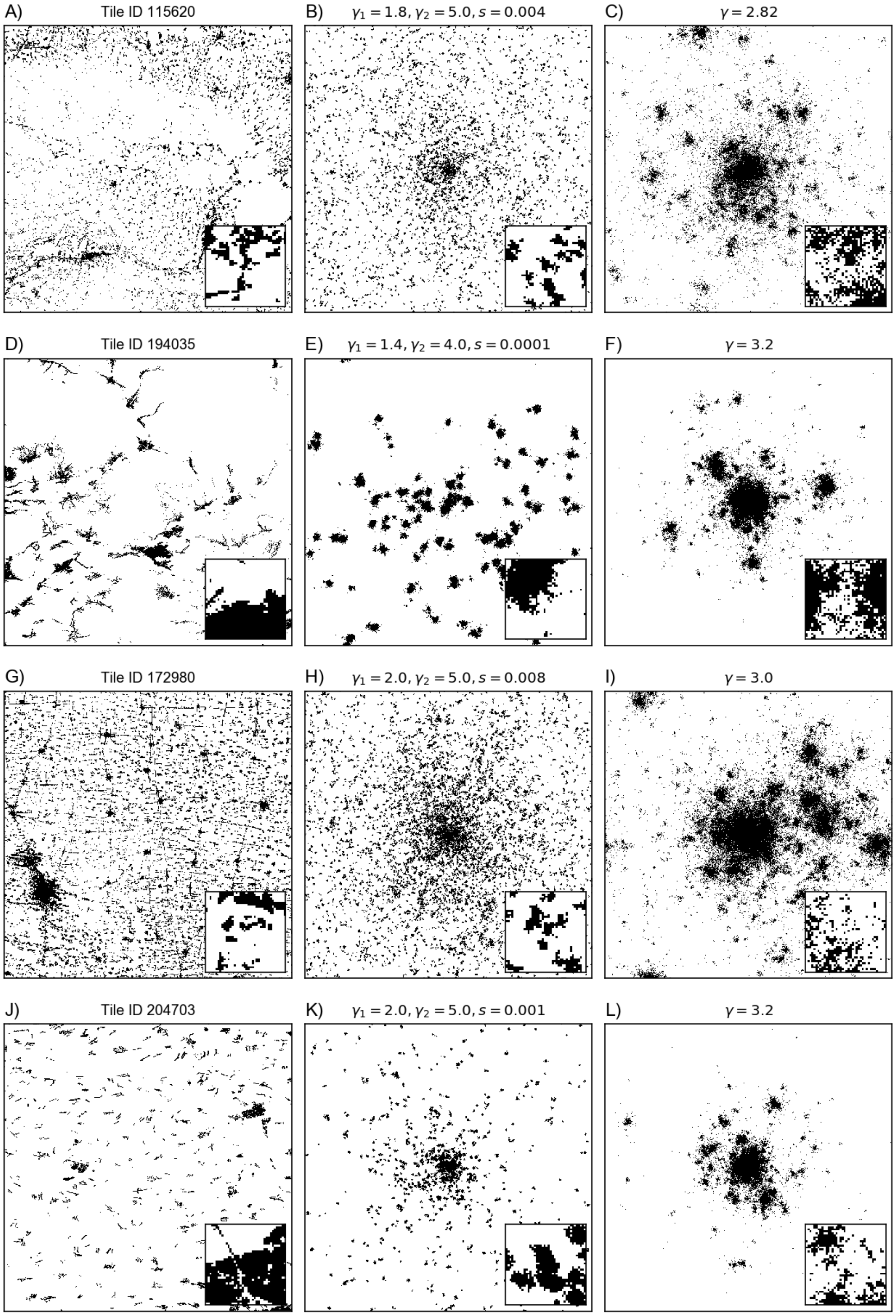

Figure S13. Some examples of the best simulations for four tiles in the Balanced class (green group). The left column shows the real tile, the central column shows the most similar tile generated with the multi-parameter model, and the right column shows the single-parameter model simulation that is most similar to the real tile.

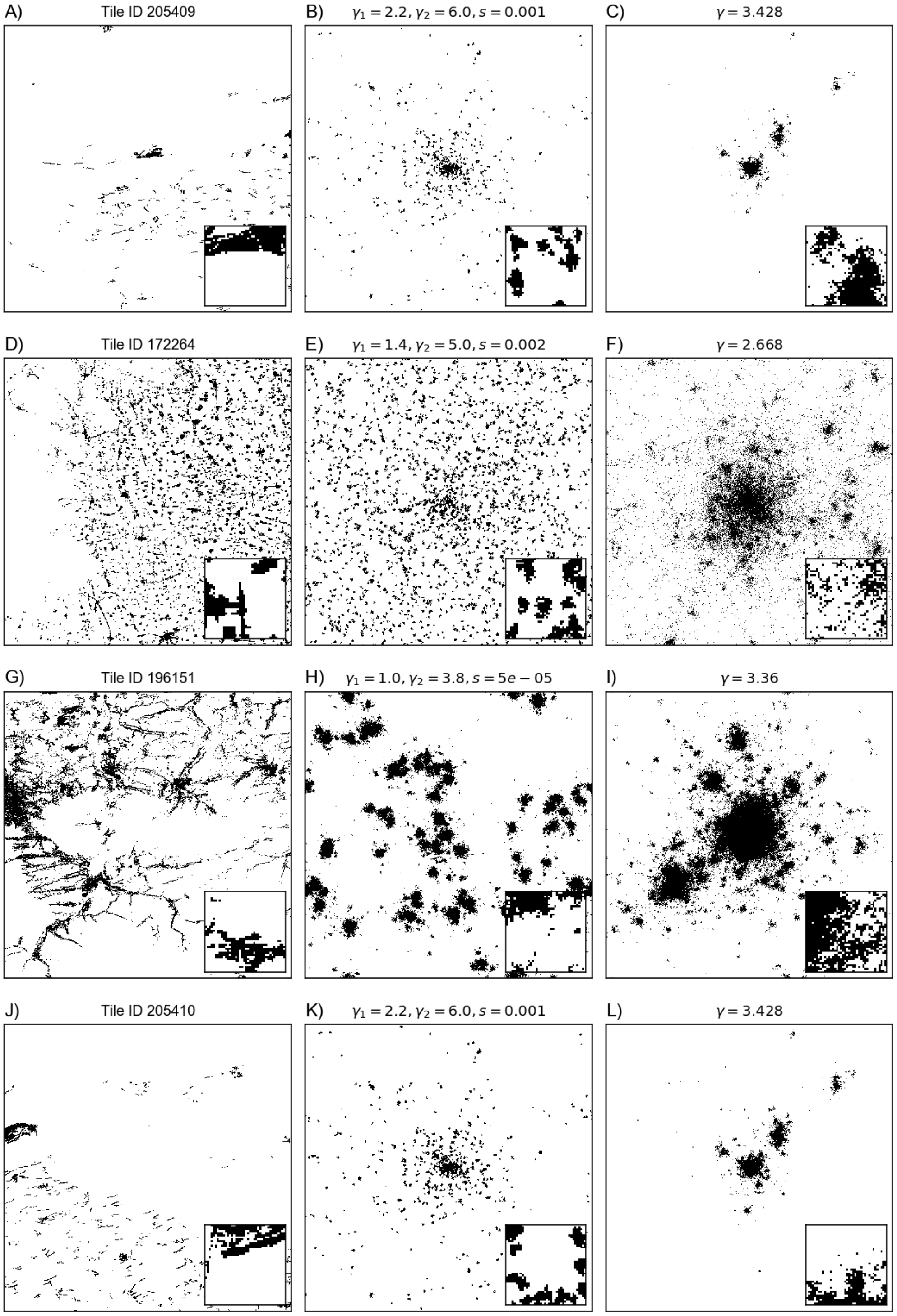

Figure S14. Some more examples of the best simulations for four tiles in the Balanced class (green group). The left column shows the real tile, the central column shows the most similar tile generated with the multi-parameter model, and the right column shows the single-parameter model simulation that is most similar to the real tile.

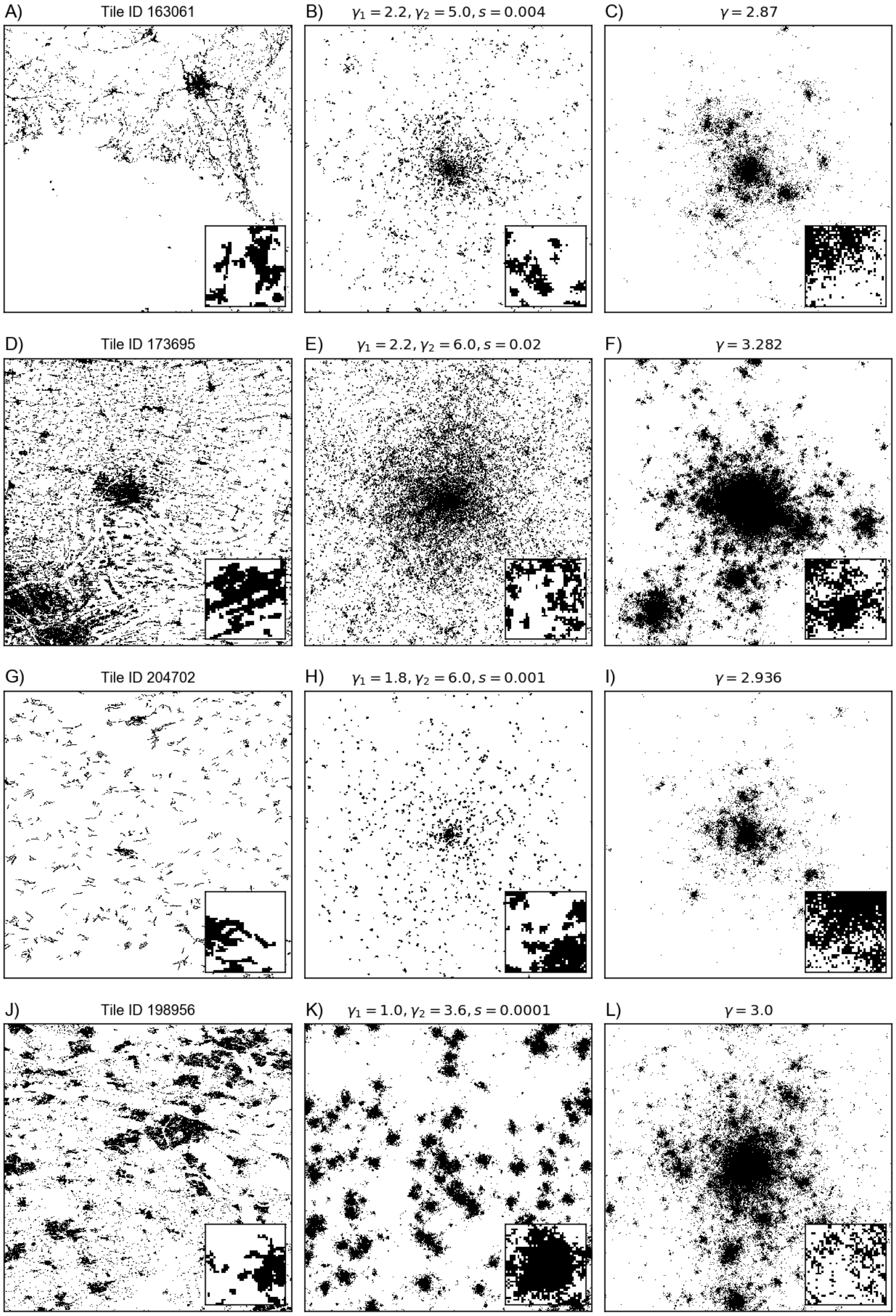

Figure S15. Some examples of the best simulations for four tiles in the Dispersion class. The left column shows the real tile, the central column shows the most similar tile generated with the multi-parameter model, and the right column shows the single-parameter model simulation that is most similar to the real tile.

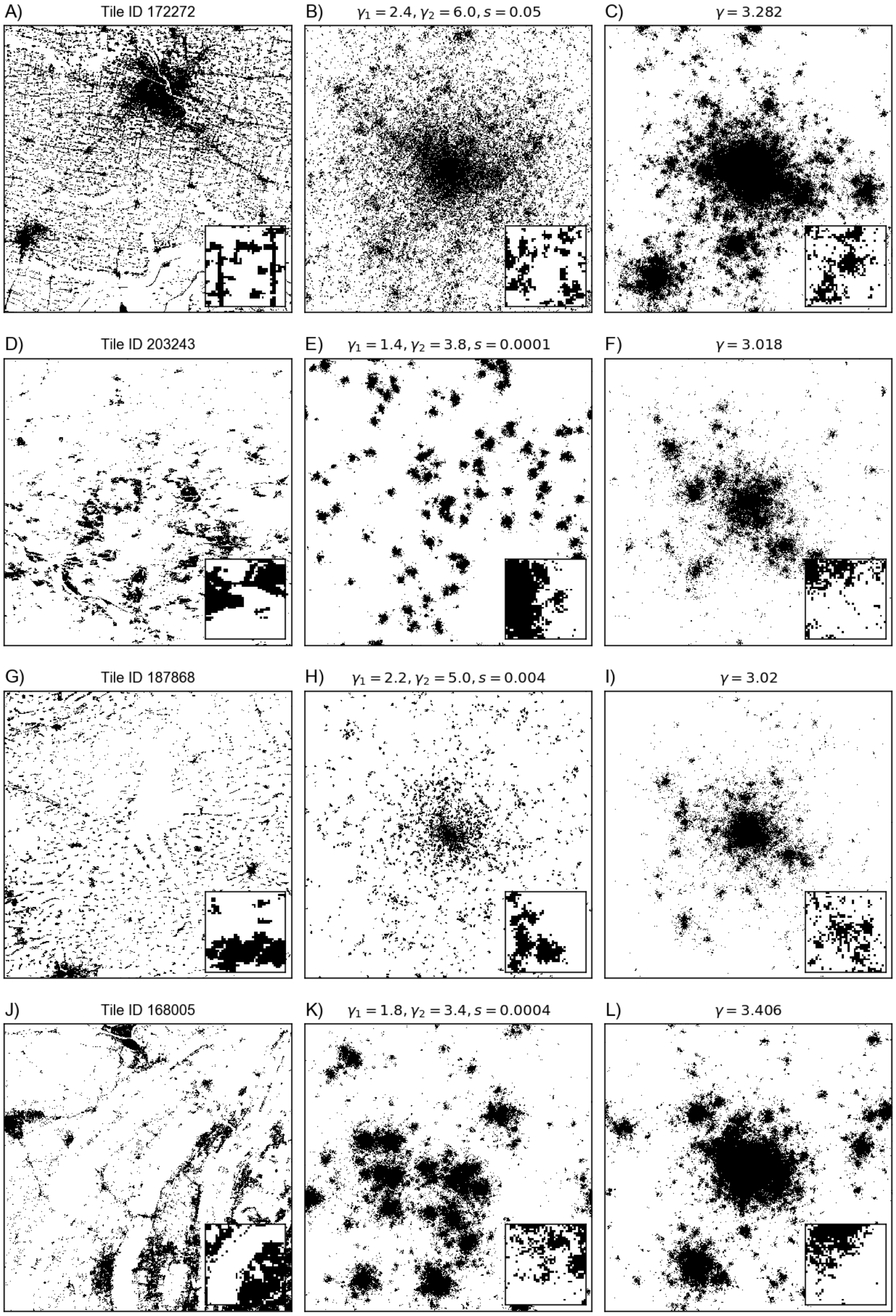

Figure S16. Some more examples of the best simulations for four tiles in the Dispersion class. The left column shows the real tile, the central column shows the most similar tile generated with the multi-parameter model, and the right column shows the single-parameter model simulation that is most similar to the real tile.

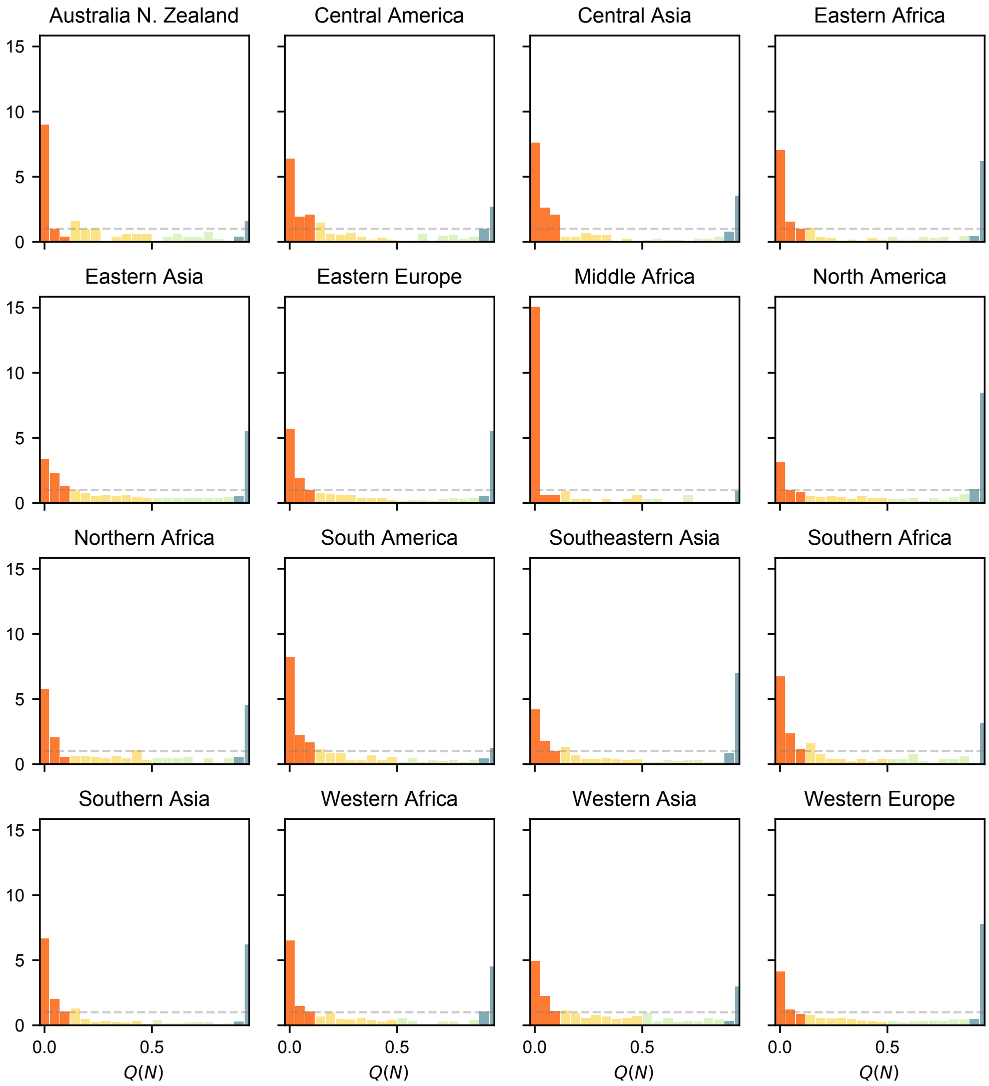

Figure S17. Agglomeration Dispersion dichotomy in multi-parameter model generated tiles.

[1] Leung, B. *et al.* Clustered versus catastrophic global vertebrate declines. *Nature* **1**, 1–5 (2020).

[2] UN. *Cities in a globalizing world: global report on human settlements 2001* (Earthscan, 2001).

[3] UN. *The state of the world cities 2004/5- Globalization and Urban Culture.* (Routledge, 2004).

[4] UN. *The state of the world cities 2006/7- The Millennium Development Goals and Urban Sustainability.* (Routledge, 2006).

[5] Birch, E. L. & S.M.Wachter. *Global urbanization* (Pennsylvania Press, 2011).

[6] Moore, M., Gould, P. & Keary, B. S. Global urbanization and impact on health. *International Journal of Hygiene and Environmental Health* **206**, 269 – 278 (2003).

[7] Zhou, L. *et al.* Evidence for a significant urbanization effect on climate in china. *Proceedings of the National Academy of Sciences* **101**, 9540–9544, DOI: 10.1073/pnas.0400357101 (2004).

[8] Kaufmann, R. K. *et al.* Climate response to rapid urban growth: Evidence of a human-induced precipitation deficit. *Journal of Climate* **20**, 2299–2306, DOI: 10.1175/JCLI4109.1 (2007).

[9] Grimm, N. B. *et al.* Global change and the ecology of cities. *Science* **319**, 756–760, DOI: 10.1126/science.1150195 (2008).

[10] Tilman, D., Balzer, C., Hill, J. & Befort, B. L. Global food demand and the sustainable intensification of agriculture. *Proceedings of the National Academy of Sciences* **108**, 20260–20264, DOI: 10.1073/pnas.1116437108 (2011).

[11] Ribeiro, H. V., Rybski, D. & Kropp, J. P. Effects of changing population or density on urban carbon dioxide emissions. *Nature communications* **10**, 1–9 (2019).

[12] Daily, G. C. & Ehrlich, P. R. Population, sustainability, and earth's carrying capacity. *BioScience* **42**, 761–771 (1992).

[13] Johnson, M. P. Environmental impacts of urban sprawl: a survey of the literature and proposed research agenda. *Environment and Planning A* **33**, 717–735 (2001).

[14] Dye, C. Health and urban living. *Science* **319**, 766–769, DOI: 10.1126/science.1150198 (2008).

[15] Seto, K. C., Fragkias, M., Güneralp, B. & Reilly, M. K. A meta-analysis of global urban land expansion. *PLOS ONE* **6**, 1–9, DOI: 10.1371/journal.pone.0023777 (2011).

[16] d'Amour, C. B. *et al.* Future urban land expansion and implications for global croplands. *Proceedings of the National Academy of Sciences* **114**, 8939–8944 (2017).

[17] Güneralp, B. *et al.* Global scenarios of urban density and its impacts on building energy use through 2050. *Proceedings of the National Academy of Sciences* **114**, 8945–8950 (2017).

[18] Herold, M., Scepan, J. & Clarke, K. C. The use of remote sensing and landscape metrics to describe structures and changes in urban land uses. *Environment and Planning A* **34**, 1443–1458 (2002).

[19] Barrington-Leigh, C. & Millard-Ball, A. A century of sprawl in the united states. *Proceedings of the National Academy of Sciences* **112**, 8244–8249 (2015).

[20] Hamidi, S. & Ewing, R. A longitudinal study of changes in urban sprawl between 2000 and 2010 in the united states. *Landscape and Urban Planning* **128**, 72–82 (2014).

[21] Huang, J., Lu, X. X. & Sellers, J. M. A global comparative analysis of urban form: Applying spatial metrics and remote sensing. *Landscape and urban planning* **82**, 184–197 (2007).

[22] Poelmans, L. & Van Rompaey, A. Detecting and modelling spatial patterns of urban sprawl in highly fragmented areas: A case study in the flanders–brussels region. *Landscape and urban planning* **93**, 10–19 (2009).

[23] Batty, M. The size, scale, and shape of cities. *Science* **319**, 769 (2008).

[24] Potere, D. & Schneider, A. A critical look at representations of urban areas in global maps. *GeoJournal* **69**, 55–80, DOI: 10.1007/s10708-007-9102-z (2007).

[25] Gamba, P. & Herold, M. *Global mapping of human settlement e Experiences, datasets, and prospects.* (CRC Press, 2009).

[26] Grekousis, G., Mountrakis, G. & Kavouras, M. An overview of 21 global and 43 regional land-cover mapping products. *International Journal of Remote Sensing* **36**, 5309–5335 (2015).

[27] Angel, S., Parent, J., Civco, D. L., Blei, A. & Potere, D. The dimensions of global urban expansion: Estimates and projections for all countries, 2000–2050. *Progress in Planning* **75**, 53–107 (2011).

[28] Pesaresi, M. *et al.* Operating procedure for the production of the global human settlement layer from landsat data of the epochs 1975, 1990, 2000, and 2014. Tech. Rep., European Join Research Center (2016).

[29] Chen, J. *et al.* 30-meter global land cover data product-globe land30. *Geomatics World* **24**, 1–8 (2017).

[30] Esch, T. *et al.* Breaking new ground in mapping human settlements from space–the global urban footprint. *ISPRS Journal of Photogrammetry and Remote Sensing* **134**, 30–42 (2017).

[31] GK, Z. *Human Behavior and the Principle of Least Effort.* (Addison-Wesley. (Cambridge, Massachusetts), 1949).

[32] Rozenfeld, H. D. *et al.* Laws of population growth. *Proceedings of the National Academy of Sciences* **105**, 18702 (2008).

[33] Gabaix, X. & Ioannides, Y. M. The evolution of city size distributions. *Handbook of regional and urban economics* **4**, 2341–2378 (2004).

[34] Rybski, D., Ros, A. G. C. & Kropp, J. P. Distance-weighted city growth. *Physical Review E* **87**, 042114 (2013).

[35] Marconcini, M. *et al.* Outlining where humans live, the world settlement footprint 2015. *Scientific Data* **7**, 1–14 (2020).

[36] Office, U. N. S. *Standard country or area codes for statistical use*, vol. 42 (UN, 1982).

[37] Gottmann, J. Megalopolis or the urbanization of the northeastern seaboard. *Economic geography* **33**, 189–200 (1957).

[38] Indovina, F., Matassoni, F. & Savino, M. *La città diffusa* (Daest Venezia, Italy, 1990).

[39] Viganò, P., Arnsperger, C., Lanza, E. C., Corte, M. B. & Cavalieri, C. Rethinking urban form: Switzerland as a “horizontal metropolis”. *Urban Planning* **2**, 88 (2017).

[40] Auerbach, F. Das gesetz der bevölkerungskonzentration. *Petermanns Geographische Mitteilungen* **59**, 74–76 (1913).

[41] Strano, E., Nicosia, V., Latora, V., Porta, S. & Barthélemy, M. Elementary processes governing the evolution of road networks. *Scientific reports* **2**, 296 (2012).

[42] Simini, F. & James, C. Testing heaps’ law for cities using administrative and gridded population data sets. *EPJ Data Science* **8**, 24 (2019).

[43] Makse, H. A., Andrade, J. S., Batty, M., Havlin, S. & Stanley, H. E. Modeling urban growth patterns with correlated percolation. *Physical Review E* **58**, 7054 (1998).

[44] Vaserstein, L. N. Markov processes over denumerable products of spaces, describing large systems of automata. *Problemy Peredachi Informatsii* **5**, 64–72 (1969).

[45] Kendall, M. G. A new measure of rank correlation. *Biometrika* **30**, 81–93 (1938).

[46] Bettencourt, L. M., Lobo, J., Helbing, D., Kühnert, C. & West, G. B. Growth, innovation, scaling, and the pace of life in cities. *Proceedings of the national academy of sciences* **104**, 7301–7306 (2007).

[47] Gomez-Lievano, A., Patterson-Lomba, O. & Hausmann, R. Explaining the prevalence, scaling and variance of urban phenomena. *Nature Human Behaviour* **1**, 1–6 (2016).

[48] Ribeiro, H. V., Oehlers, M., Moreno-Monroy, A. I., Kropp, J. P. & Rybski, D. Association between population distribution and urban gdp scaling. *Plos one* **16**, e0245771 (2021).

[49] Zhou, B. *et al.* A gini approach to spatial co2 emissions. *Plos one* **15**, e0242479 (2020).
